# Genome Wide Association Study of Seedling and Adult Plant Leaf Rust Resistance in Elite Spring Wheat Breeding Lines

**DOI:** 10.1371/journal.pone.0148671

**Published:** 2016-02-05

**Authors:** Liangliang Gao, M. Kathryn Turner, Shiaoman Chao, James Kolmer, James A. Anderson

**Affiliations:** 1 University of Minnesota, Department of Agronomy and Plant Genetics, St. Paul, MN, 55108, United States of America; 2 USDA-ARS Cereal Disease Laboratory, St. Paul, MN, 55108, United States of America; 3 University of Minnesota, Department of Plant Pathology, St. Paul, MN, 55108, United States of America; 4 USDA-ARS Biosciences Research Lab, Fargo, ND, 58102, United States of America; Mahatma Phule Agricultural University, INDIA

## Abstract

Leaf rust is an important disease, threatening wheat production annually. Identification of resistance genes or QTLs for effective field resistance could greatly enhance our ability to breed durably resistant varieties. We applied a genome wide association study (GWAS) approach to identify resistance genes or QTLs in 338 spring wheat breeding lines from public and private sectors that were predominately developed in the Americas. A total of 46 QTLs were identified for field and seedling traits and approximately 20–30 confer field resistance in varying degrees. The 10 QTLs accounting for the most variation in field resistance explained 26–30% of the total variation (depending on traits: percent severity, coefficient of infection or response type). Similarly, the 10 QTLs accounting for most of the variation in seedling resistance to different races explained 24–34% of the variation, after correcting for population structure. Two potentially novel QTLs (QLr.umn-1AL, QLr.umn-4AS) were identified. Identification of novel genes or QTLs and validation of previously identified genes or QTLs for seedling and especially adult plant resistance will enhance understanding of leaf rust resistance and assist breeding for resistant wheat varieties. We also developed computer programs to automate field and seedling rust phenotype data conversions. This is the first GWAS study of leaf rust resistance in elite wheat breeding lines genotyped with high density 90K SNP arrays.

## Introduction

Wheat leaf rust, caused by the fungus *Puccinia triticina* Eriks, is a threat to world wheat production. Identification of resistance genes using molecular markers is an important step toward marker assisted selection and resistance breeding. To date, there have been more than 70 leaf rust resistance genes identified, the majority of which confer leaf rust resistance in the seedling stage and are race-specific [1]. Identification of resistance loci or major QTLs that confer adult plant or field resistance against leaf rust will enhance our ability to develop leaf rust resistant wheat varieties.

One way to identify leaf rust resistance QTLs, is through association mapping (AM). Association mapping has the potential of accommodating wide collections of germplasm and due to historic recombination, AM studies generally have better mapping resolution compared to bi-parental mapping [2]. With decreasing sequencing costs and the advent of high throughput genotyping assays [3–5], association mapping using high density genome wide markers, also known as genome wide association study (GWAS), has become more widely available to plant researchers.

Association mapping for various traits in a number of crop species, including rice, corn, soybean, wheat, barley, tomato and potato have been conducted [2, 6]. Association mapping studies were also conducted for various rust disease traits in wheat [7–13]. Maccaferri et al [7] reported association mapping of leaf rust resistance using mostly SSR markers and a collection of durum wheat *(Triticum turgidum* L. var. *durum*, tetraploid AABB), but not common wheat (allohexaploid AABBDD). Kertho et al [13] explored resistance QTLs in a collection of winter wheat landraces, but the study focused on seedling resistance, with no data on field resistance. Few GWAS studies were conducted for mapping leaf rust resistance loci in both seedling and adult plant stages, and in elite bread wheat cultivars or breeding lines. Furthermore, to our knowledge, no study has utilized the recently developed high density iSelect 90K SNP array [5] for GWAS analysis of leaf rust resistance in bread wheat.

The main feature of this mapping panel is that a large amount of the germplasm possesses resistance to leaf rust and in some cases specific genes providing the resistance are known. It is possible that such a panel will allow us to identify resistance alleles that are normally not detected due to low allele frequency. This study aims to validate known genomic loci effective to leaf rust resistance and to identify novel genes or QTLs that are effective against the leaf rust pathogen in the seedling and (or) adult plant stages. Meanwhile, this study also explores the genetic architecture and phenotypic correlations for seedling and adult plant resistance and discusses ways to implement our research results in plant breeding and genetics efforts.

## Materials and Methods

### Plant Materials

A total of 381 spring wheat breeding lines derived from commercial and public breeding sectors were selected to form a leaf rust association mapping (AM) panel. These lines were from different countries of North, Central and South America, as well as some parts of Africa, Europe and Asia. Public sector contributors included the University of Minnesota (MN), North Dakota State University (NDSU), South Dakota State University (SDSU), the United States Department of Agriculture, Agricultural Research Service (USDA-ARS), International Center for Maize and Wheat Improvement (CIMMYT), Uruguay, Argentina, Brazil, and Chile as well as some European and Asian countries. Private sector contributors included Syngenta (Basel, Switzerland), Limagrain (Puy-de-Dome, France) and Westbred (Monsanto Co, St. Louis, MO). The AM panel consists of wheat lines with leaf rust resistance to one or multiple races. Over 70 Thatcher near isogenic lines (NILs) [14] with one or more known leaf rust resistance genes were also included to assist with gene postulations.

### Leaf Rust Disease Phenotyping

The 381 plants were grown under greenhouse conditions and inoculated at the first leaf seedling stage, 7d after planting, in three independent experiments, each using a different race or combination of races. Races of *P*. *triticina* included race 1 BBBDB (Long and Kolmer 1989) which is widely avirulent to many Lr genes in wheat; race CA1.2 (BBBQD), a race that is virulent to most durum wheat cultivars and avirulent to most bread wheat cultivars; and a mixture of six races of MHDSB, MFPSB, MLDSB, TBBGJ, TFGJQ and TFBGQ common in North America. The six race mixture was also used to inoculate plants in the field. Disease phenotypes were scored 10–12 d after inoculation, according to the 0 to 4 infection type (IT) scale developed by Stakman et al. [15]. The phenotype data was converted to a linearized 0 to 9 scales using a custom Perl script and analyzed using R [16] to derive summary statistics. The program for seedling rust score conversion adopted the scales developed by Zhang et al [10] with slight modifications. Specifically, if it is a simple reading such as “1+” or “2-”, the original 0–9 scale proposed by Zhang et al was used; if it is a complex reading such as “;13+”, the readings were first split into simple readings such as “;” “1” “3+”, then the first reading was weighted double, all readings were converted to 0–9 scales, and arithmetic means were calculated. The program is fully automated and can take data tables with practically unlimited number of columns or trait values. With slight modifications, this program can be used to convert other types of text based categorical data into numeric scales (https://github.com/umngao/rust_scores_conversion).

The 381 lines (with four check cultivars Thatcher, Tom, Verde and Knudson) were planted in single rows in the field and inoculated with the race mixture (see above), approximately one month after planting when the entries were starting to tiller. A mixture of the susceptible cultivars Max, Little Club, Thatcher and Morocco were planted perpendicular to the entries and were inoculated with the race mixture to uniformly infect the entries. Phenotyping (rust scoring) was done approximately one month post inoculation after anthesis on flag leaves of adult plants. Phenotypes were collected in four years and two locations (2012 in Crookston, MN and 2012–2015 in St. Paul, MN). Leaf rust data was scored using the percentage of diseased leaf area (“Field.SEV” or severity) using a modified Cobb scale [17] and response (“Field.IT” or infection type) based on pustule sizes of uredinia spores and amount of necrosis and chlorosis, on a scale of resistant to susceptible response that was converted to a numeric 0 to 1 scale [18]. The phenotypic data were automatically converted to three measures (severity, response and coefficient of infection) using another custom Perl script for field rust score conversion (https://github.com/umngao/rust_scores_conversion), whereas “severity” (Field.SEV) = percentage of diseased leaf area; “response” (Field.IT) = a numeric scale of 0 to 1. It is worth noting that field. IT was converted to a 0–1 linearized scale, while seedling infection types against race1, race.CA1.2 or race.Mix were converted to a 0–9 linearized scales [18]. Coefficient of infection (Field.COI) equals to the product of “severity” and “response”. All of these measures were used in association mapping, with COI values being the primary trait for selection of most significant QTLs, as it combines the information from both severity and response or IT for field resistance.

Phenotypic values were further adjusted based on a mixed linear model, with environments (each year and location combination was counted as one environment for a total of five environments: CrK12, StP12, StP13, StP14, StP15) as random effects, and genotypes as having fixed effects. Best linear unbiased estimates (BLUE) combined the trait values across years. Heading and flowering dates were also collected and initially included into the mixed model analysis. Because significant effects of heading or flowering on overall leaf rust disease severity for this particular dataset were not detected, they were later dropped from the model. Mixed model analysis was performed using the “lme4” package of the open source statistical language or software environment R [16].

### DNA Extraction and Genotyping

DNA was collected from seedling plants using a Qiagen (Venlo, Limberg, Netherlands) Biosprint method, and genotyped with the Illumina’s iSelect 90K SNP array at the USDA-ARS Biosciences Research lab in Fargo, North Dakota, USA. Forty-two of the 381 lines were phenotyped, but not genotyped, thus not included into GWAS analysis. The data generated were called and curated using Illumina GenomeStudio software, and uploaded to the T3 database (http://triticeaetoolbox.org/wheat/). SNPs were filtered and converted to Hapmap format using a custom Perl script incorporating the criteria for: minor allele frequency (MAF) greater than 0.05; missing data less than 20% (only two lines had missing data greater than 15%) and chromosome positions previously mapped in the wheat 90K consensus map [5]. A molecular marker (*csLV34*) [19] tightly associated to *Lr34*, was used to genotype this panel, and the genotyped results were included in GWAS analysis.

### Population Structure (Q), Kinship (K) and Linkage Disequilibrium (LD) Analysis

Population structure (Q) was analyzed using a model based clustering method (admixture models with correlated allele frequencies) in STRUCTURE [20] v2.3.4, and principal component analysis (PCA) using the statistical software R. An LD-based pruning method implemented in the PLINK software [21] v1.09 was used to prune the total filtered marker set (18,924) using the command line option of “—indep-pairwise 100 5 0.2” under Linux environment. The pruned (or filtered) set of SNP markers (1309) were used for structure analysis. The reason for using pruned markers rather than the whole set of markers is that STRUCTURE assumes loci are at linkage equilibrium within sub-populations.

Ten independent STRUCTURE runs were conducted for each specified K (number of subpopulations, from 2 to 10), with 20,000 burn-in length and 40,000 Markov chain Monte Carlo (MCMC) iterations under Linux environment. The most likely number of clusters (K) was chosen based on the ΔK method [22], implemented in a web-based informatics tool “Structure Harvester” [23]. The method estimates ΔK based on the rate of change in the log probability between successive K values. Clumpp [24] software v1.1 was used to consolidate STRUCTURE runs and derive the Q matrix used in AM with mixed linear models (MLM). DISTRUCT v1.1 [25] was used to plot the population Q matrix bar graph. The fixation index (*Fst*) of subpopulations was obtained through STRUCTURE run outputs.

TASSEL [26] v4.3.13 was used to derive a population Kinship matrix based on the scaled IBS (identity by state) method using the complete set of markers that passed quality filtering. Previous research has shown that using the complete set of markers is useful to control genome-wide error rate and performs much better than pedigree based methods for kinship estimates [27]. Linkage disequilibrium (LD) was calculated using TASSEL. LD decay curves for each sub-genome (A, B, D) were fitted using a non-linear model described by Marroni et al. [28]. The LD values (R^2^) were also used to assist QTL block determination (see next section for details). Some LD blocks or adjacent regions were selected using PLINK software tool set [21] and visualized using Haploview software [29].

### Genome Wide Association Study (GWAS) Using MLM (QK), QGLM and G-Model Analysis

Association mapping based on mixed linear model (MLM) and generalized linear model with population structure as a covariate (QGLM) analysis was conducted primarily using TASSEL [26], a JAVA based open source software for linkage and association analysis. MLM model results were further validated using the Genome Association and Prediction Integrated Tool (GAPIT) [30] under open source R environment. The GAPIT tool does not report QGLM values. We also explored the use of a Fortran software or program developed by Dr. Rex Bernardo (University of Minnesota) for GWAS analysis using genome wide markers to control for background variations in a multiple regression setting (known as G-model) (http://bernardo-group.org/books-and-software/) [31]. For this study, we focused primarily on MLM results from TASSEL outputs, but the results from GAPIT and G-models were used in a supporting manner.

The Mixed linear model (MLM) approach [32] was used in GWAS analysis of leaf rust resistance. The mixed linear model for GWAS can be specified as follows: y = Xβ + Qv + Zu +e, where y is the phenotype values (either BLUE adjusted or environment specific values), β and v are fixed effects due to marker and population structures respectively, u is a vector of random effects due to the portion of breeding values not accounted for by the markers. X, Q and Z are incidence matrices that related y to β, v and u. The covariance matrix of breeding value “u” is the product of kinship matrix (commonly designated as A or K) and Vg, the portion of additive variance that is not accounted for by the marker under test [33]. Considering the size of the population in this study, we did not use the “compressed MLM” ability of GAPIT or Tassel which groups individuals based on phenotypes [34].

P-values, R^2^ and marker effects were extracted from GWAS results. The false discovery rate (FDR) adjusted p-values used in GAPIT were found to be highly stringent. Researchers have debated that correcting for marker effects based on both population structure Q and kinship K (which in turn were often calculated based on marker data as well) could be over-correcting and might result in a need for relaxed p-value levels such as 0.001 [11, 35] or alternative ways to correct for background variations [31]. Some researchers have developed or adopted alternative multiple test correction methods such as “*simpleM*”, a variant of the Bonferroni correction [36, 37]. The *SimpleM* [36] approach applies a Bonferroni correction to the effective number of independent tests (*Meff*). In this study, marker–wise significance was based on three criteria (p<0.05, p<0.001, and *SimpleM*) with increasing stringency. For QTL nomination, a p-value of less than 0.001 (detected in at least one environment) was required. For count-based analysis of previously designated QTLs using vennCounts and vennDiagram functions from LIMMA (linear model for microarrays, a Bioconductor package of R), a p-value cutoff of 0.05 was used to capture the global similarity or dissimilarity between traits or environments. An effective number of tests (*Meff*) were calculated for each chromosome individually and the *SimpleM* level was determined as p-value = 7.72E-5, derived from 0.1 divided by the total number of *Meff* for all chromosomes (n = 1295).

R^2^ values from LD analysis, in conjunction with genetic distances were used to assign co-segregating or adjacent significant markers into a unique QTL block based on the criteria of R^2^ value greater than 0.2 and genetic distance less than 10 cM, with a few exceptions: QTLs 1B_1 and 4A_3 both had markers that are more than 10 cM apart, yet were almost completely linked (LD R^2^≈1).

### Most Significant Markers, Stepwise Regression, and Postulation of Gene Names

For each LD block or QTL as defined above, markers with the most significant p-values or largest R^2^ were extracted as representative markers for each marker-trait association. The most significant 10 QTLs for each trait (field disease resistance, seedling resistance against Race 1, CA1.2, and the race mixture) were summarized in more detail and the cumulative effects of significant markers were assessed using a multiple regression approach (considering additive effects only) with population structure as a covariate.

Stepwise regressions were performed on significant markers to identify the loci that were independently associated with disease resistance for each trait, respectively, with population structure Q as covariate. For field BLUE trait values, a p-value cutoff of 0.005 was required for inclusion in the stepwise regression (the selected markers also passed the 0.001 threshold for at least one of the tested environments). For seedling traits, if less than 10 loci passed the 0.001 threshold (e.g. QTLs resistant to race CA1.2), the most significant 10 loci were used, roughly equivalent to a p-value cutoff of 0.1 percentile. For both forward and backward selection (in stepwise regression of the multiple regression models), a p-value of 0.05 determined whether including or dropping a given QTL from the model was appropriate.

The most significant QTLs were postulated based on position, LD, infection type and pedigree or donor line information. All sequences associated with 90K SNPs were blasted against the wheat genome survey sequences v2 (http://www.wheatgenome.org/) using command line version of BLAST+ v2.2.8 under Linux environment. A few target SNP sequences were also used to BLAST against a newer version of wheat genome build [38] and reference Barley genome sequences [39] for candidate gene annotation. The criterion was percent identity greater than 95% and only the best hit was taken if there were multiple BLAST hits. The BLAST hit information was used to explore candidate genes for certain QTLs and to assist optimal chromosome arm assignments for certain SNPs. For this study, candidate genes or QTLs were postulated or designated for those loci whose p-values (derived from either MLM or QGLM analysis) surpassed the *simpleM* Bonferroni correction threshold. The candidate QTLs identified through G-model generally have higher significance levels (strongly exceeding the *simpleM* threshold).

## Results

### Phenotype Data and Results

**Fig 1:**
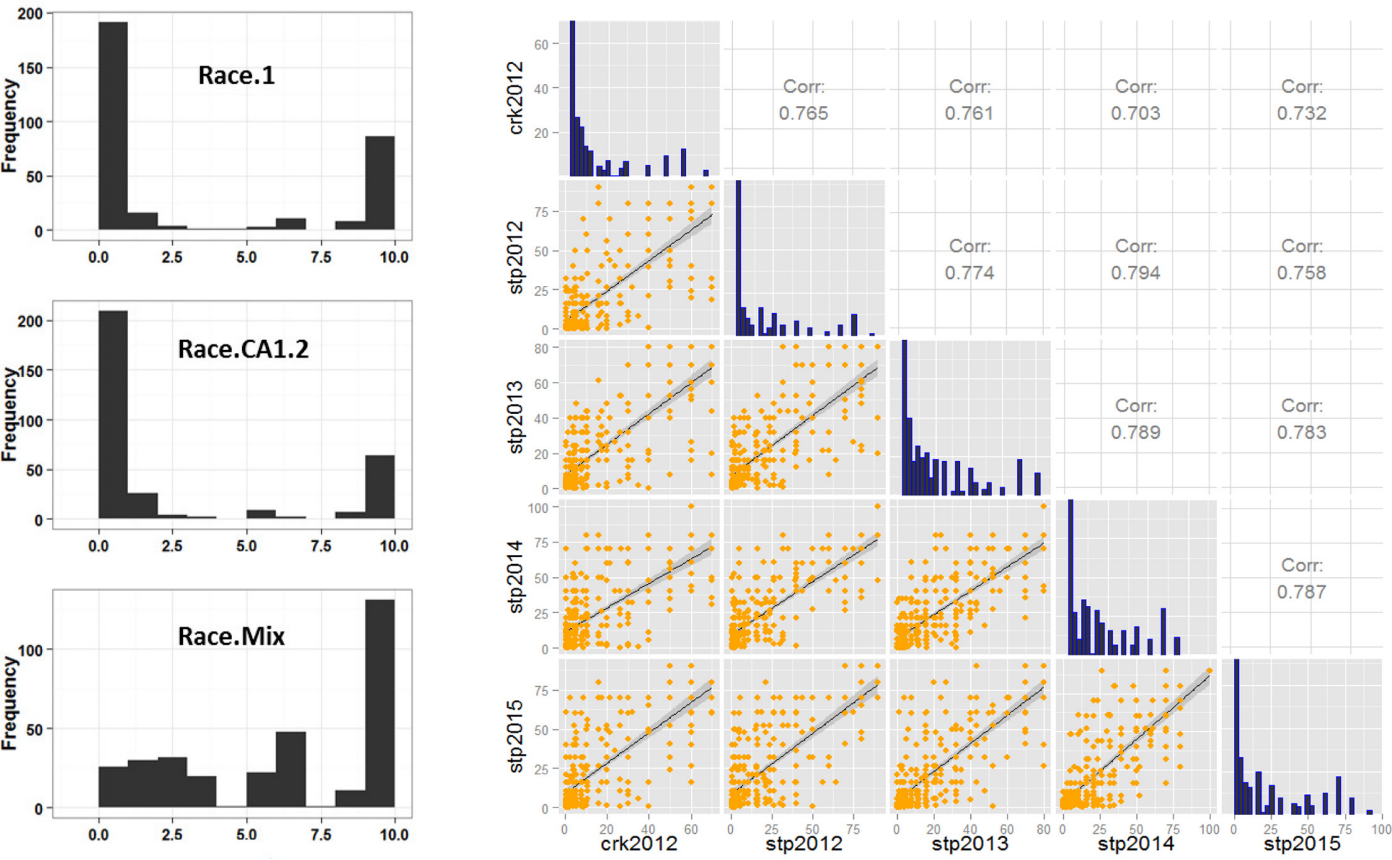
Seedling disease levels and field disease (coefficient of infection, COI) correlations. (a) Left (black and white) panel showing seedling disease distributions; X-axis represents linearized 0–9 scales; (b) Right (orange and blue) panel showing field disease correlations. Diagonals are histogram for each environment (Crookston CRK12,Saint Paul StP12-15).

For seedling plants, more than 50% of the tested lines were completely resistant (immune, with IT = 0) against leaf rust race 1 or race CA1.2 (Fig 1). This suggests the presence of resistance genes in many of the tested lines. Approximately 70% of the highly resistant lines are resistant to both race 1 and race CA1.2, likely suggesting that these lines possess major genes that are effective against both races. The median seedling leaf rust disease score was 0 (the completely immune type) for resistance to race 1 and CA1.2 and 6 (equivalent to IT of 2+) for resistance to the field race mixture. The mean disease severity of the race mixture was higher than the two single races by 2.8 and 3.4 respectively, on a 0–9 disease scale. The ANOVA analysis for treatment effects (or inoculation method) p-value was less than 2.2E-16. The higher disease severity for race mixtures was expected, as the field race mixtures contain races that are more virulent than race 1 and race CA1.2. Nonetheless, approximately 40% of the tested lines were resistant (with IT of 2+ or less) to the field race mixtures at the seedling stage, suggesting that these lines are broadly resistant to common North American races and also may be resistant in field trials.

Disease severity varied between years of field plot tests, with the year 2012 displaying the lowest leaf rust severity. Nonetheless, the year to year correlations are still high, with an average Pearson correlation coefficient of R = 0.77 (Fig 1), similar to those observed for a stripe rust study [40]. After a mixed model adjustment, we estimated the heritability (h^2^) of leaf rust disease trait (for coefficient of infection, COI) to be 0.92. The overall disease distribution among the 381 lines was highly skewed toward resistance types (Fig 1B, diagonal histograms). Coefficient of infections (COI) for 69% of the lines was less than 20 (on a 0–100 scale). These results suggest that most of the breeding lines used in this AM study contain effective resistance genes or QTLs against multiple leaf rust races under field environments.

The overall Pearson’s correlations among measured traits are listed in Table 1. Seedling plant infection types to single races (race 1 and race CA1.2) were moderately correlated with each other (0.63). We also observed a moderate correlation between seedling and field response (IT) to race mixtures (R = 0.57). Our results showed a high correlation (R> = 0.88) among field trait values calculated using different methods (COI, severity, and response or infection type IT). The field response and response to single races are less well correlated (R<0.29), which was expected as race 1 and CA1.2 are highly avirulent, while the mixture of race used for the seedling and field plot tests is virulent to a number of Lr genes.

**Table 1:** Phenotype correlations among field and seedling traits (Race.1, Race.CA1.2, Race.Mix, Field.COI, FIELD.SEV, FIELD.IT).

	Race.1	Race.CA1.2	Race.Mix	Field.COI	Field.SEV	Field.IT	
**Race.1**	-						
**Race.CA1.2**	0.63	-					
**Race.Mix**	0.41	0.31	-				
**Field.COI**	0.20	0.29	0.50	-			
**Field.SEV**	0.18	0.25	0.51	0.98	-		
**Field.IT**	0.17	0.22	0.57	0.90	0.88		-

### Genome Wide SNP Coverage and Linkage Disequilibrium (LD) Based on 90K SNP Arrays

A total of 18,925 markers passed the quality filters (18,924 of them are SNP array based markers). On average, there are 1.4 markers per centimorgan (cM) (S1 Fig). Marker density on D genome chromosomes was much lower compared to A and B genomes: one marker every 2.6 cM compared to one marker every 0.74–0.97 cM. This is roughly proportional to the total mapped markers for each genome [5].

**Fig 2:**
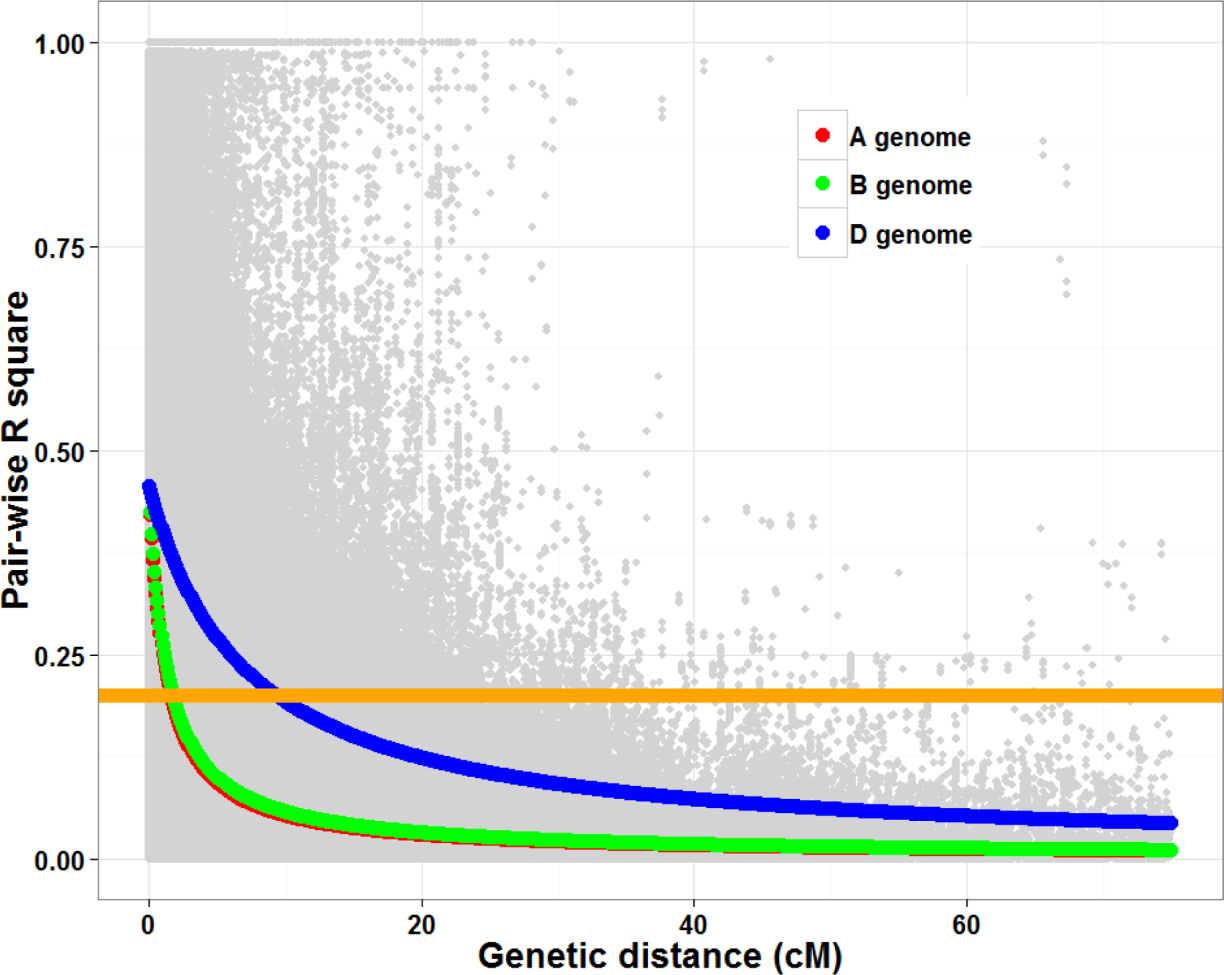
Linkage disequilibrium based on 18924 SNP markers. For color fitted LD decay lines: red represents A genome; green represents B genome; blue represents D genome. Orange bar indicates LD decay level (R2 = 0.2).

We found that the LD decays (as defined by R^2^ declining to below 0.2) at around 1.5–1.7 cM for A and B genomes, but extends to more than 8 cM in D genome chromosomes (Fig 2), consistent with previous findings [5]. The high LD in D genome chromosomes has the practical effect of reducing the number of markers required to detect significant marker traits associations.

### Population Structures of the Leaf Rust Association Mapping Panel and Correlation with Leaf Rust Phenotype in the Field

**Fig 3:**
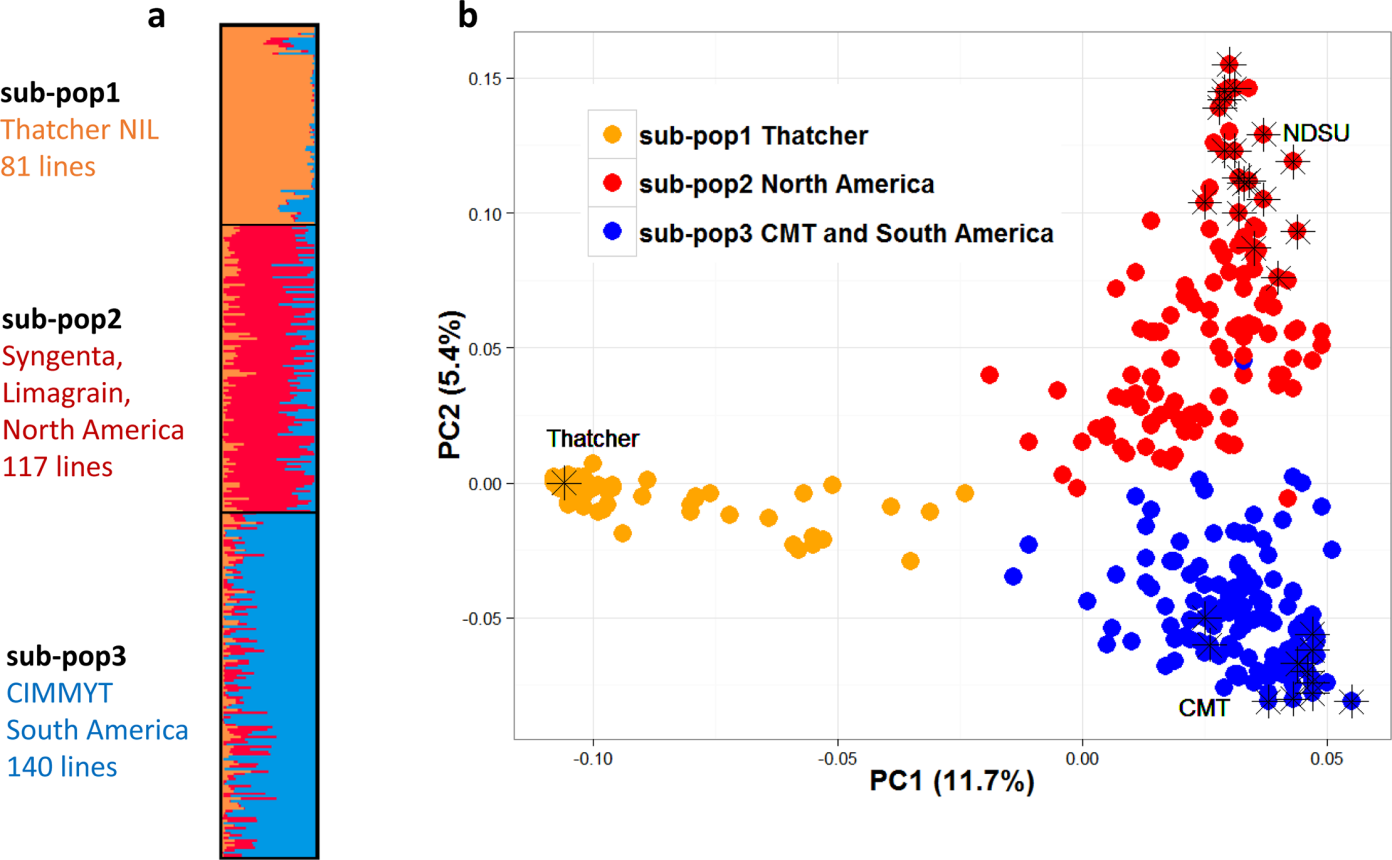
Population structure: model based approach and PCA approach. (a) model based approach for population structure analysis. (b) PCA approach for population structure analysis. Orange: sub-population 1; Red: sub-population 2; Blue: sub-population 3. Asterisks (*) on PCA plot indicate accessions that are typical of each sub-population. Sub-population 3 also has lines from North America. Both sub-pop2 and sub-pop 3 might include lines from Asia, Europe etc.

For model based analysis using STRUCTURE [20], the optimal number of K was determined to be 3 based on the ΔK method [22]. In this study, ΔK value equals 380 for K = 3 and the remaining tested K’s all have a much smaller ΔK value (mean = 2, max = 5). Similarly, for PCA based analysis, we found that the total variance explained by each principal component (PC) drops sharply for the first three PCs (from 11.7 to 5.4 to 3.3), and levels after PC3. The first three PCs capture over 20% of the total variance. Given the results from both model based and PCA analysis, we used K = 3 for constructing the Q matrix in the mixed model marker trait association analysis in both TASSEL and GAPIT. The population structure derived from the model–based and PCA–based approaches were very similar (Fig 3). GWAS results (identified QTLs and p-values for markers) using different version of Q3 matrices (either model based or PCA based) agreed with each other: The correlation among sets of p-values and marker effects were both over 0.9 for the field BLUE trait, and the correlation among set of R^2^ values was over 0.96 for the same trait (Field.COI).

**Fig 4:**
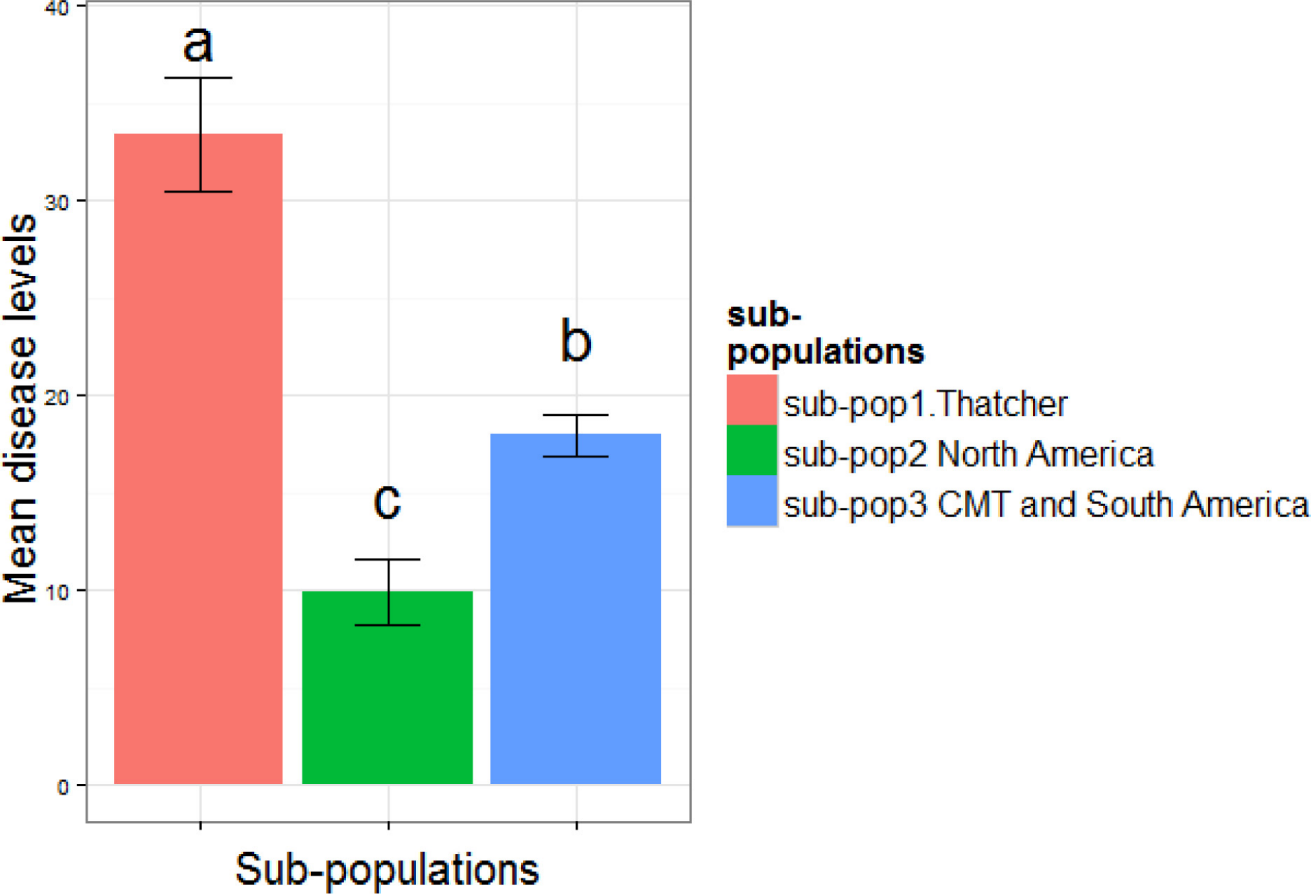
Different sub populations of this AM panel are associated with different levels of leaf rust disease severity (measured using coefficient of infection Field.COI).

STRUCTURE analysis revealed three sub-populations: i) Sub-population 1 (81 lines) consisted of almost entirely Thatcher near isogenic lines (NILs) with a few exceptions; ii) Sub-population 2 (117 lines) consisted of mostly (~70%) North American lines including genotypes from South Dakota State University, University of Minnesota, North Dakota State University, Syngenta and Limagrain; iii) The majority (with only one exception) of CIMMYT and South American lines were in sub-population 3 (140 lines). However, sub-population 3 also contained some North American lines. A few European, African and Asian lines were also present in Sub-populations 2 and 3. The fixation index (Fst) values for the three sub-populations are 0.80, 0.43 and 0.12 respectively, suggesting that sub-population 3 has a much higher divergence within itself. Different sub-populations are associated with different resistance levels and the differences are highly significant (p < 1.8E-5). Sub-population 1 (Thatcher near isogenic lines) has the highest disease (mean 33.4), followed by sub-population 3 (South America, mean 18.0) and sub-population 2 (North America, mean 9.9) (Fig 4).

### Marker Trait Associations by MLM (QK), QGLM and G-Model Analysis

After trimming the genotype and phenotype datasets, 18,925 markers and 338 lines were included in the GWAS analysis. As an exploratory analysis, GWAS (MLM) for plant flowering date phenotype analysis revealed a single genomic locus at 90cM (long arm) of chromosome 5A using GAPIT with default settings (S2 Fig). The marker trait association p-value was highly significant (p < 1E-10). We hypothesize that this locus is *Vrn-A1*, consistent with previous mapping results [4]. The successful detection of *Vrn-A1* suggests that the MLM (with population structure Q and kinship K) model fits the data. We further utilized quantile-quantile (QQ) plots to examine the MLM model fitness for leaf rust traits. The expected–log_10_P value and the observed–log_10_P value distributions follow the X = Y diagonal line until it curves at the end (S3 Fig). These results further suggest that our MLM models fit the data.

After fitting the MLM using TASSEL [26], a total of 333 markers were identified to be significantly (p < 0.001) associated with leaf rust resistance for at least one of the field or seedling traits (S1 Table) We combined adjacent markers based on LD and genetic distance information, and were able to obtain 46 unique QTLs (Table 2).

**Table 2:** A total of 46 QTLs were identified that were significantly associated with field or seedling leaf rust disease resistant (p.MLM < 0.001).

QTL (gene)^a^	Chr	Position (90K)	Num SNPs	Crk 2012	StP 2012	StP 2013	StP 2014	StP 2015	Lr.COI	Lr.SEV	Lr.IT	Race.1	Race CA1.2	Race Mix
1A_3	1A	148.99–151.22	6	4.43E-03	6.89E-05	2.01E-03	8.55E-05	6.34E-04	1.11E-04	8.63E-04	2.35E-03	-	-	3.55E-03
1B_1	1B	43.66–64.89	88	1.17E-02	3.39E-04	2.09E-03	6.23E-04	2.56E-03	1.07E-03	2.01E-03	4.78E-05	-	9.21E-03	3.77E-04
1B_t1	1B	81.95–82.86	2	1.96E-02	7.36E-04	5.27E-03	8.63E-04	8.03E-03	1.62E-03	1.17E-03	1.49E-02	9.03E-03	-	-
1B_2	1B	84.43–85.57	11	-	-	-	-	-	-	-	-	7.84E-05	1.96E-02	-
1D_1	1D	3.5–8.71	4	1.46E-02	7.02E-03	4.90E-03	5.87E-03	7.72E-03	1.80E-03	3.55E-03	1.31E-04	-	1.05E-02	1.46E-03
1D_2	1D	44.69–44.69	2	-	-	9.64E-03	-	1.48E-02	1.76E-02	2.67E-02	4.48E-04	-	-	-
1D_t1	1D	45.44–45.44	1	-	-	-	-	-	-	-	-	3.85E-04	-	-
1D_3	1D	88.85–89.58	5	2.45E-02	-	5.10E-03	1.49E-02	-	1.44E-02	2.81E-02	-	2.97E-02	3.96E-04	-
2A_1	2A	20.14–20.14	1	-	-	-	-	-	-	-	-	3.64E-02	-	2.58E-04
2A_2	2A	101.97–101.97	1	-	-	-	-	3.28E-04	3.66E-02	-	1.61E-02	-	2.21E-02	-
2A_3	2A	108.46–108.46	1	1.69E-02	4.64E-04	3.69E-03	1.78E-03	1.19E-02	4.38E-04	1.54E-03	2.60E-03	3.94E-02	5.61E-03	-
2B_2	2B	88.44–97.26	24	3.45E-06	1.95E-02	2.88E-03	7.75E-03	1.20E-04	4.52E-04	5.97E-04	7.80E-04	-	9.08E-05	4.47E-02
2B_3	2B	102.28–108.35	15	1.57E-03	4.29E-04	4.61E-03	3.45E-04	1.75E-03	7.55E-04	1.38E-03	2.13E-03	-	-	-
2D_1	2D	18.22–18.22	1	-	-	6.82E-03	1.12E-02	9.26E-03	4.84E-03	3.48E-04	5.73E-04	-	-	-
3A_1	3A	86.16–87.78	3	-	8.17E-04	9.32E-04	5.56E-05	9.54E-03	1.50E-03	3.72E-04	4.15E-02	-	-	3.11E-02
3A_t2	3A	169.89–169.89	1	1.25E-03	1.50E-03	4.96E-03	2.96E-03	1.73E-03	4.31E-04	7.47E-04	1.19E-03	-	-	1.16E-03
3B_t2	3B	51.07–51.08	2	3.09E-02	-	5.14E-03	3.50E-02	1.32E-02	1.82E-02	-	1.34E-03	-	-	6.69E-04
3B_1	3B	139.62–139.62	3	-	1.29E-02	4.62E-05	4.14E-02	-	8.23E-03	3.06E-02	3.53E-02	-	-	-
4A_1 (umn-4AS)	4A	37.05–37.05	2	7.72E-03	9.45E-04	8.60E-05	3.50E-04	2.20E-03	4.17E-05	4.74E-05	9.64E-05	3.24E-02	-	-
4A_2	4A	48.52–48.84	60	1.34E-02	-	7.26E-05	-	2.34E-02	1.69E-02	4.00E-02	3.22E-02	-	6.04E-03	2.89E-02
4A_3	4A	51.7–53.13	7	1.39E-03	1.24E-03	8.79E-03	1.18E-04	3.01E-02	6.67E-04	1.40E-03	3.69E-03	-	-	-
4A_t1	4A	73–73	1	2.57E-02	6.18E-03	2.89E-02	5.72E-04	1.31E-02	1.43E-03	9.18E-03	2.34E-03	-	-	-
4A_t2	4A	139.97–144.38	4	-	3.09E-02	4.26E-02	7.16E-03	1.53E-02	1.41E-02	4.17E-03	3.24E-04	5.59E-04	7.94E-03	-
4B_1	4B	15.91–15.91	1	-	-	1.91E-02	-	6.01E-03	7.47E-03	9.71E-03	7.71E-04	-	-	4.02E-03
4B_3	4B	74.62–90.07	10	5.64E-05	2.77E-03	1.73E-03	1.99E-04	1.07E-02	1.56E-04	1.02E-04	1.35E-03	-	2.44E-02	2.93E-03
4D_1	4D	70.59–70.59	1	7.58E-03	6.42E-03	5.06E-03	6.95E-03	1.35E-02	1.82E-03	2.78E-03	2.26E-04	2.34E-02	1.90E-02	2.31E-04
5B_1	5B	39.4–39.4	8	-	1.67E-03	5.73E-03	2.41E-03	4.05E-04	9.90E-04	1.51E-03	4.03E-02	-	-	-
5B_2	5B	49.01–49.65	2	6.71E-04	8.99E-04	2.34E-02	5.95E-06	2.07E-03	1.69E-04	6.78E-04	1.14E-03	5.08E-05	-	-
5B_t1	5B	119.54–119.54	1	9.68E-04	3.26E-02	2.05E-02	-	-	3.58E-02	3.46E-02	-	-	-	-
5D_1 (*Lr1*)	5D	203.88–204.58	6	-	-	-	-	-	-	-	-	1.17E-04	3.74E-02	-
6A_1	6A	25.86–27.15	2	4.60E-04	-	2.22E-02	8.55E-03	7.57E-03	1.32E-03	5.59E-03	3.16E-03	-	-	-
6A_t1	6A	48.09–48.09	1	4.38E-03	4.46E-03	1.70E-02	9.81E-03	8.01E-04	4.97E-04	5.57E-04	3.28E-03	-	-	-
6A_2	6A	100.62–100.62	1	-	-	-	-	-	-	-	-	2.23E-04	1.24E-02	-
6A_3	6A	119.64–119.64	1	1.56E-02	-	-	-	-	-	-	-	-	-	2.28E-04
6B_1	6B	16.76–16.76	1	-	-	-	-	-	-	-	-	9.43E-04	-	-
6B_3	6B	66.36–66.36	2	-	8.10E-03	-	3.04E-03	2.84E-02	1.36E-02	1.24E-02	1.14E-02	2.33E-03	5.05E-04	7.02E-04
6B_4 (*Lr3*)	6B	118.99–122.92	31	-	-	-	-	-	-	-	-	6.02E-07	8.78E-05	8.81E-03
7A_3	7A	125.47–125.47	1	9.59E-04	2.90E-02	-	-	3.24E-02	1.25E-02	2.01E-02	-	-	-	-
7B_1	7B	58.17–58.63	3	-	3.60E-04	6.11E-04	7.56E-03	2.32E-02	1.57E-02	2.54E-02	2.51E-03	-	4.03E-03	-
7B_2	7B	66.62–71.33	8	-	-	-	-	-	-	-	-	-	1.22E-04	-
7B_t1	7B	73.79–73.79	1	4.73E-02	1.47E-02	1.59E-02	2.77E-02	9.50E-03	3.88E-03	2.53E-03	5.60E-04	-	-	-
7B_3	7B	76.31–76.31	2	-	-	-	-	-	-	-	-	2.09E-04	-	-
7B_t2	7B	89.13–89.13	2	1.90E-02	9.54E-03	3.54E-03	-	6.00E-04	2.60E-03	4.48E-03	3.74E-03	-	-	-
7B_4	7B	166.99–166.99	1	1.89E-04	2.58E-04	7.93E-04	1.42E-03	2.35E-03	5.03E-04	1.66E-03	6.36E-03	-	-	-
7D_1 (*Lr34*)	7D	35.00–35.00^b^	1	8.16E-05	7.55E-03	2.15E-02	-	3.15E-02	3.51E-03	2.03E-03	-	-	3.22E-02	-
7D_2	7D	169.51–169.51	1	2.86E-03	1.15E-02	-	-	6.53E-04	2.91E-03	3.91E-03	2.32E-02	-	-	-

^a^Postulated loci names are given (within parentheses) if strong evidences to support such postulations were obtained.

^b^The approximate *Lr34* position on the 90K map is based on blast hit information on a recent chromosome 7D scaffold build published by Chapman et al. [38].

Columns “CrK2012”, “StP2012”- “StP2015” indicate GWAS p-values for individual field environments in Crookston and St Paul, MN.

Columns “Lr.COI”, “Lr.SEV”, “Lr.IT” indicate GWAS p-values for BLUE estimated field traits (coefficient of infection, severity and infection type).

Underlined p-values are those that surpass the *simpleM* Bonferroni correction threshold.

**Fig 5:**
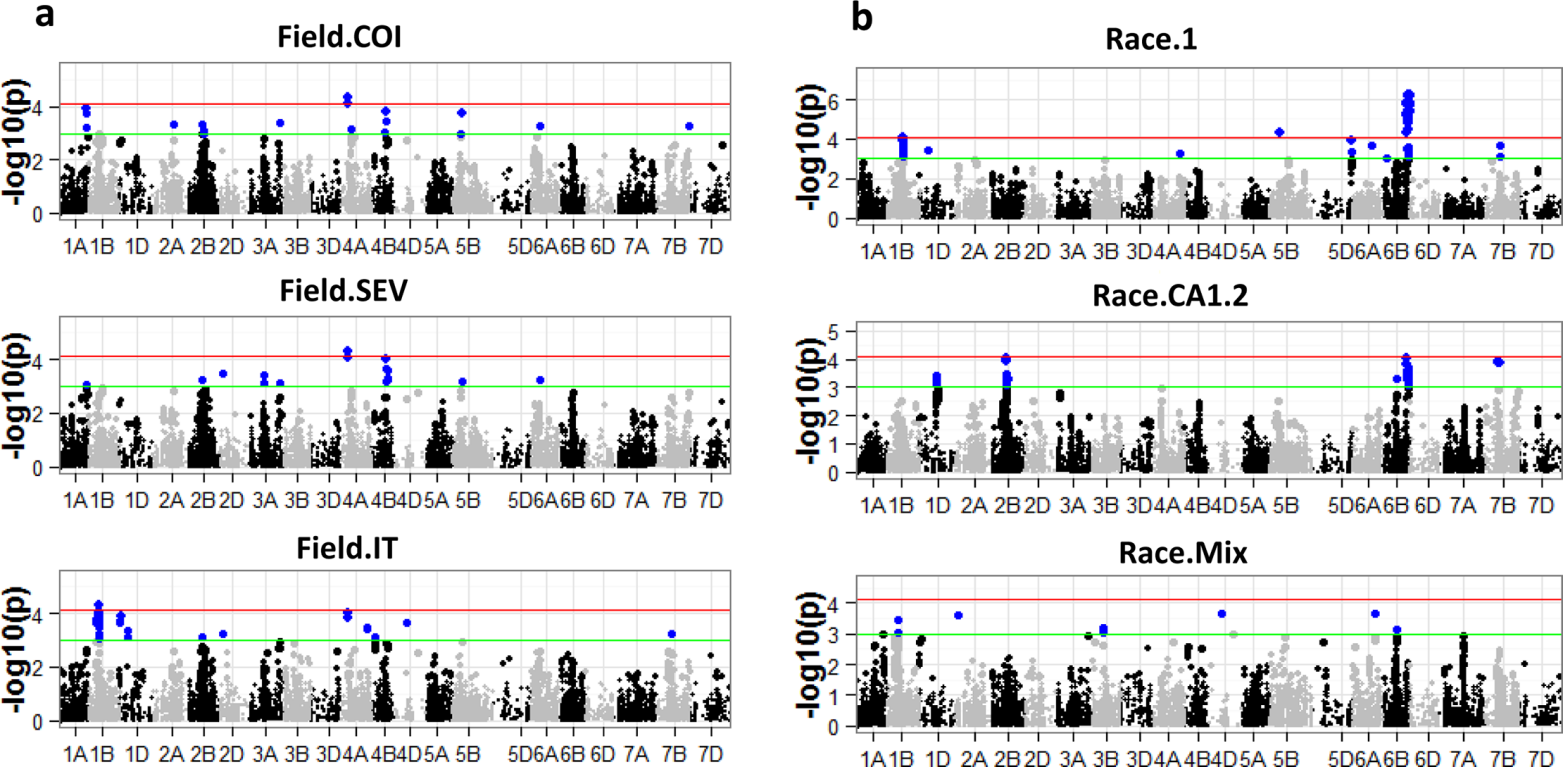
Manhattan plots for field (a) and seedling (b) traits. Red line indicates *SimpleM* bonferroni corrected p-value threshold for significance (7.7x10-5); Green line indicates p-value of 1x10-3.

**Fig 6:**
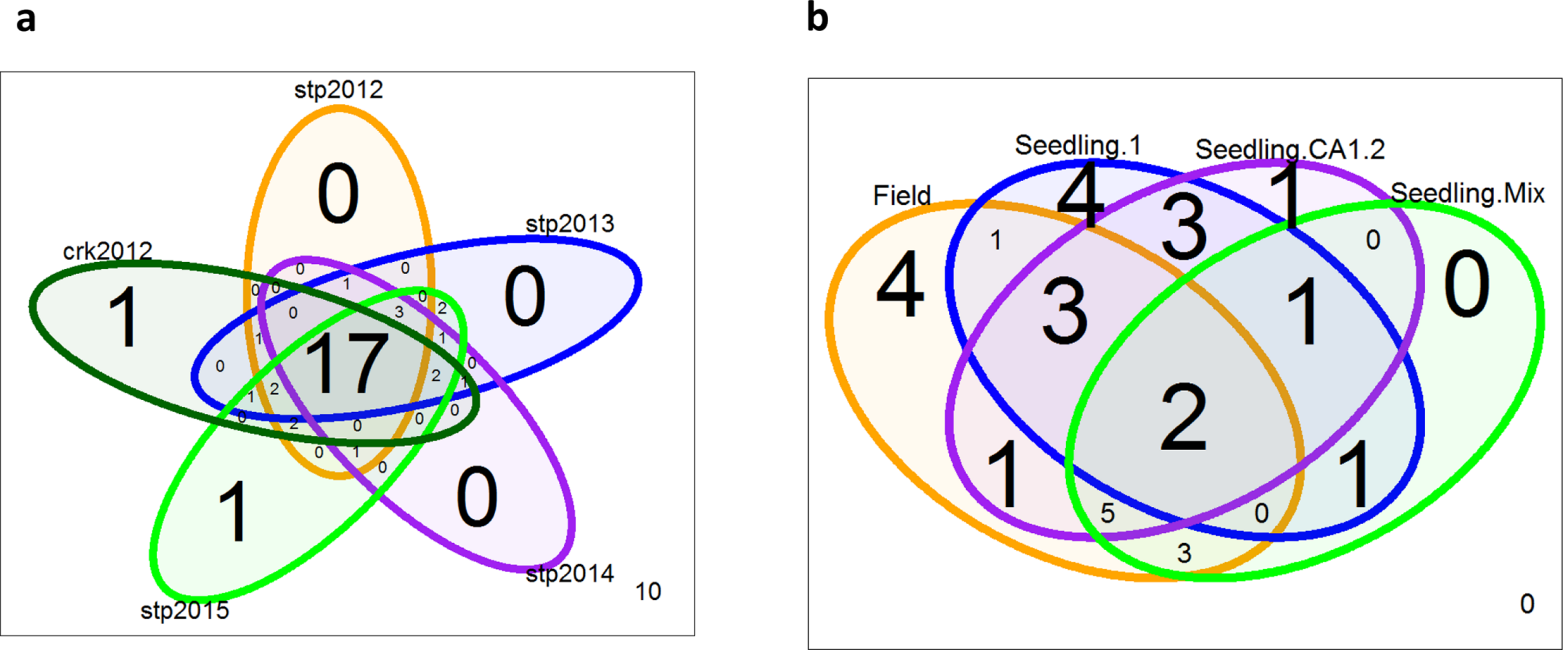
Venn diagrams (a) Overlap between QTLs under different field environments. (b) Overlap between field and seedling QTLs.

Manhattan plots for the BLUE estimated field traits across different field environments (2012–2015) are shown in Fig 5. A majority of the field QTLs were detected under multiple environments (Table 2 and Fig 6). Similar to MLM analysis, exploratory analysis using QGLM model (population structure + generalized linear model) also revealed the known flowering locus at 5AL (data not shown). The QGLM p-values for leaf rust traits are generally more significant compared to MLM analysis (Table 3).

**Table 3:** Most significant (top 10) QTLs and representative SNPs for field and seedling traits.

Trait	QTL	Marker	blast	Chr	Position	p.MLM	p.QGLM	RSQ	eff	R	Sub pop1	Sub pop2	Sub pop3	postulation	reference
Race.1	1B_2	**IWB13336**	NA	1B	85.57	7.84E-05	**1.39E-06**	0.05	2.76	C	78	99	108	Lr26?	Kolmer 2003
Race.1	1D_t1	**IWB14612**	1DS_1885467	1D	45.44	3.85E-04	**2.77E-05**	0.04	2.48	A	77	107	101	Lr42?	Liu et al 2013
Race.1	4A_t2	IWB3569	4AL_v2_7176180	4A	144.38	5.59E-04	**1.08E-05**	0.04	2.32	A	10	107	83	Lr28?	Bipinraj et al 2011
Race.1	5B_2	**IWB9055**	NA	5B	49.65	**5.08E-05**	**1.44E-06**	0.06	2.35	G	76	101	78	QLr. cdl-5BL?	Kolmer 2015
Race.1	5D_1	**IWB53861**	NA	5D	203.88	1.17E-04	**2.32E-05**	0.05	-2.58	A	3	16	30	Lr1	Kolmer 2003
Race.1	6A_2	IWB625.2	NA	6A	100.62	2.23E-04	**5.76E-06**	0.05	1.97	G	75	57	87	Lr64?	-
Race.1	6B_1	**IWB65148**	NA	6B	16.76	9.43E-04	**4.82E-06**	0.04	2.51	G	9	114	102	-	-
Race.1	6B_3	**IWB65914**	6BL_4378239	6B	66.36	2.33E-03	3.06E-04	0.03	1.83	G	43	110	112	Lr9?	Kolmer 2003
Race.1	6B_4	**IWB3292**	6BL_4278271	6B	122.92	**6.02E-07**	**1.18E-07**	0.09	-3.43	C	4	14	23	Lr3	Kolmer 2003
Race.1	7B_3	**IWA306**	7BL_6747122	7B	76.31	2.09E-04	**9.56E-07**	0.05	3.16	G	75	115	115	Lr14?	Kolmer 2003
Race.CA1.2	1B_1	**IWB19584**	NA	1B	63.91	9.21E-03	3.24E-04	0.02	-2.46	G	4	3	20	QLr.cimmyt-1BS	Rosewarne 2012
Race.CA1.2	1D_3	**IWB35520**	1DL_2290849	1D	89.58	3.96E-04	**5.16E-08**	0.04	2.42	A	79	113	94	Lr21?	
Race.CA1.2	2A_3	**IWA3151**	2AL_6426630	2A	108.46	5.61E-03	**2.97E-05**	0.03	-1.40	T	4	49	104	Lr11.Lr38?	Darino 2015
Race.CA1.2	2B_2	**IWB37811**	2BS_5186722	2B	93.47	9.08E-05	**8.75E-08**	0.05	1.91	G	73	47	82	Lr13.Lr23.Lr16?	Oelke & Kolmer 2005
Race.CA1.2	4A_2	IWB40915	4AS_v2_6008166	4A	48.52	6.04E-03	1.13E-04	0.03	1.95	T	79	116	95	-	-
Race.CA1.2	4A_t2	IWB3569	4AL_V2_7176180	4A	144.38	7.94E-03	1.86E-04	0.02	1.57	A	10	107	83	Lr28?	Bipinraj et al 2011
Race.CA1.2	6B_3	**IWB65914**	6BL_4378239	6B	66.36	5.05E-04	**1.16E-06**	0.04	1.89	G	43	110	112	Lr9?	Kolmer 2003
Race.CA1.2	6B_4	**IWB6474**	NA	6B	119.73	8.78E-05	**1.53E-05**	0.06	2.27	G	9	110	95	Lr3	Kolmer 2003
Race.CA1.2	7B_1	IWB39492	7BS_3168118	7B	58.17	4.03E-03	1.60E-02	0.03	1.76	A	72	111	113	Lr72?	Herrera-Foessel et al. 2014
Race.CA1.2	7B_2	**IWB68484**	7BS_3079273	7B	66.62	1.22E-04	**3.19E-07**	0.05	4.06	T	80	111	126	Lr14?	Singh et al. 2009
Race.Mix	1A_3	**IWB48030**	1AL_3976804	1A	149.82	3.55E-03	5.99E-04	0.03	1.72	G	10	113	103	QLr.umn-1AL	*
Race.Mix	1B_1	IWB19584	NA	1B	63.91	3.77E-04	**2.67E-07**	0.05	-3.44	G	4	3	20	QLr.cimmyt-1BS	Rosewarne et al 2012
Race.Mix	1D_1	IWB44021	1DS_1912623	1D	8.71	1.46E-03	**9.51E-07**	0.04	-3.22	T	4	3	17	Lr42?	Liu et al 2013
Race.Mix	2A_1	**IWB74529**	NA	2A	20.14	2.58E-04	**4.56E-06**	0.05	2.10	C	79	36	121	Lr17?	Kolmer 2003
Race.Mix	3A_t2	**IWB34789**	3AL_4449581	3A	169.89	1.16E-03	**3.57E-05**	0.04	1.57	G	8	61	105	QLr.fcu-3AL?	Chu et al. 2009
Race.Mix	3B_t2	**IWB74350**	3B_10762316	3B	51.07	6.69E-04	8.56E-05	0.04	1.74	A	73	83	85	-	-
Race.Mix	4B_3	IWB72129	4BL_7035179	4B	86.55	2.93E-03	**4.75E-06**	0.03	1.55	G	76	80	103	Lr30?	Draz et al 2015
Race.Mix	4D_1	**IWB17540**	4DS_2288313	4D	70.59	2.31E-04	**1.78E-07**	0.05	-3.35	T	6	3	19	?	
Race.Mix	6A_3	IWA7764	6AL_5772638	6A	119.64	2.28E-04	**1.13E-06**	0.06	2.12	C	74	60	83	-	-
Race.Mix	6B_3	**IWB11702**	6BL_4398818	6B	66.36	7.02E-04	**5.60E-05**	0.04	1.86	T	43	111	125	Lr9?	Kolmer 2003
Field.COI	1A_3	**IWB48030**	1AL_3976804	1A	149.82	1.11E-04	**7.23E-06**	0.05	12.98	G	10	113	103	QLr.umn-1AL	*
Field.COI	2A_3	**IWA3151**	2AL_6426630	2A	108.46	4.38E-04	**1.17E-06**	0.04	-10.14	T	4	49	104	Lr11?	Darino et al 2015
Field.COI	2B_2	**IWB22236**	2BS_5202128	2B	88.44	4.52E-04	**6.65E-06**	0.04	10.02	T	13	79	104	Lr13.Lr23.Lr16?	Oelke & Kolmer 2005
Field.COI	3A_t2	**IWB34789**	3AL_4449581	3A	169.89	4.31E-04	**8.19E-06**	0.04	9.48	G	8	61	105	QLr.fcu-3AL?	Chu et al. 2009
Field.COI	4A_1	**IWB59410**	4AS_v2_5925149	4A	37.05	**4.17E-05**	**7.73E-06**	0.05	11.23	T	7	74	99	QLr.umn-4AS	*
Field.COI	4A_3	IWB7998	NA	4A	51.7	6.67E-04	1.11E-04	0.04	-9.63	T	38	38	16	-	-
Field.COI	4B_3	IWB7278	4BL_6967384	4B	78.96	1.56E-04	**6.47E-06**	0.04	13.46	T	81	108	103	QLr.cimmyt-4BL?	William et al 2006
Field.COI	5B_2	**IWB39735**	5BL_10794137	5B	49.01	1.69E-04	**6.69E-06**	0.04	20.17	C	80	115	124	QLr. cdl-5BL?	Kolmer 2015
Field.COI	6A_t1	IWB40242	NA	6A	48.09	4.97E-04	3.26E-04	0.04	-11.69	T	4	10	61	-	-
Field.COI	7B_4	IWB64015	7BL_6699942	7B	166.99	5.03E-04	9.98E-05	0.04	10.87	T	75	107	81	Lr68	Herrera-Foessel et al 2012

Column "Marker", underlining indicates SNPs detected by or significant in stepwise regression models.

Columns "p.MLM" and "p.QGLM" reflect p-values from the MLM and QGLM methods, underlining indicates p-values passing the *simpleM* threshold.

Column R indicates resistance (or favorable) allele.

Columns “RSQ” and “eff” indicate marker R^2^ and effects based on MLM models.

Columns “Sub pop1”, “Sub pop2” and “Sub pop3” indicate number of favorable allele within each sub-populations. Column "postulation" are postulated genes or QTLs based on position, infection type, and donor parents or pedigree information. Some of the loci names were adopted from Li et al [41]. The postulated gene or QTLs followed by questions marks "?" are primarily based on position and infection type (pedigree or donor line information was unobtainable).

Column "reference" shows literature reports that support our loci postulation. Dash "-" signs indicate that the corresponding p-values are below the *simpleM* threshold and loci identity were not postulated. Asterisks "*" indicate potentially novel loci identified through this study.

We used the GAPIT tool to validate QTLs detected by TASSEL. A total of 44 out of 46 QTLs detected by TASSEL (96%) were also detected using GAPIT (S2 Table). By relaxing the p-value threshold slightly from 0.0010 to 0.0013, all of the 46 QTLs (100%) were significantly detected by GAPIT (S2 Table). We also explored the use of G-model [31] to do GWAS analysis, the p-value used in multiple regression fitting is 0.000001 (highly significant and far surpassing the simpleM threshold), and a total of 108 markers were identified for the Field.COI trait. Many of these markers share similar positions with those identified through MLM and QGLM analysis (S3 Table). Overall, our GWAS results showed high agreement across multiple environments and between various methods of QTL detection.

### Gene or QTL Postulations for the Most Significant (Top 10) QTLs for Seedling and Field Resistance

Combining the most significant 10 QTLs (based on MLM p-values, abbreviated as p.MLM) for each trait (Race1, Race.CA1.2, Race.Mix, Field.COI) resulted in a total of 29 unique QTLs. Some of the loci were detected for multiple traits, thus the unique number of QTLs for the four traits is 29 instead of 40 (Table 3). We found that a number of loci were likely known or previously identified loci that are involved in rust resistance. For example, QTL 5D_1 (203.88 cM) located on long arm of chromosome 5D (Table 3), approximately the same location as *Lr1*. This locus was detected only in the seedling stage, which is consistent with *Lr1* being not effective to races present in the field (environments in which the locus was not detected were represented with a dash “-” in Table 2). Thatcher near isogenic line RL6003 (known to possess *Lr1*) has the favorable allele. This evidence suggests that QTL 5D_1 is *Lr1*. One of the most significant loci for seedling resistance against race 1 and CA1.2 was QTL 6B_4, which was located on long arm of chromosome 6B (122.92cM, Table 3, Fig 5). Thatcher near isogenic lines RL6002 and RL6042 were known to possess *Lr3* [42], and both of these lines possess the favorable SNP allele (C). This evidence suggests that QTL 6B_4 is *Lr3*. Our data show that the *Lr68* representing contig 7BL_6748067 [43] was positioned at 171 cM based on BLAST hit information, roughly at the same location of QTL 7B_4 (166.99 cM). QTL 7B_4 is effective at the adult plant stage only (Table 2). All these results suggest that QTL 7B_4 (SNP IWB64015) represents *Lr68*.

Besides *Lr1*, *Lr3*, *Lr68*, a number of quantitative trait loci (Table 2) were mapped to approximately the locations of loci *Lr26* (QTL 1B_2) [29], *Lr42* (1D_t1) [44], *Lr21* (1D_3), *Lr17*(2A_1), [42], *Lr11* (2A_3) [45], *Lr28* (4A_t2) [46], *Lr14* (7B_3) [47], QLr.cimmyt-1BS (1B_1) [48], QLr.cdl-5BL (5B_2) [49], and possibly *Lr30* and others [50, 51]. However, due to the lack of pedigree and donor line genotype information (within this panel), and also the near fixation of some alleles within sub-population 1 (i.e., majority of Thatcher NILs have the same allele), more genetic experiments are needed to further confirm these postulations, especially for those that are postulated based primarily on position.

We also designated two potentially novel loci (Table 3) that are associated with different seedling and field rust traits with p-values surpassing the *simpleM* threshold (7.72E-5). QTL 1A_3 (*QLr*.*umn-1AL*) was mapped to position 149.8 cM on chromosome 1AL for both field BLUE and seedling race mixture traits (Table 3). Chromosome 1AL is not associated with adult leaf rust resistance [41]. It is also not likely to be *Lr59* [52] because *Lr59* is located on an alien introgression that is unlikely to be present in this AM panel. QTL 4A_1 (*QLr*.*umn-4AS*), highly associated with field resistance (both p.MLM and p.QGLM surpassing the *simpleM* threshold), was represented by two SNP markers (IWB13323 and IWB59410), located at 37 cM of 4AS (Fig 5 and Table 2). No known QTLs or genes are present on chromosome 4AS that confer resistance to leaf rust.

### Trait Genetic Architecture Revealed by Most Significant QTLs and by Loci Selected Using Stepwise Regression Models

The top 10 QTLs for field resistance were all detected in at least three field environments (p.MLM < 0.001), or all of the five environments (CrK12, StP12, StP13, StP14, StP15) when using less stringent p.MLM cutoffs of 0.02 (S1 Table and Table 2). Our results also show that QTLs associated with the seedling race mixture trait had a higher overlap with QTLs detected in the field (Fig 6, Table 2), consistent with the finding that the two traits are moderately correlated (R > 0.5, Table 1). QTL 1B_1 (likely QLr.cimmyt-1BS, Table 3) is highly effective (p.MLM = 3.77E-4, p.QGLM = 2.67E-7) against seedling race mixtures, but is not among the most significant ones for BLUE estimated coefficient of infection (COI) trait (Field.COI), despite a moderately significant p-value (p.MLM = 1.07E-3) (Table 2). Interestingly, it is the most significant QTL for field response type (Field.IT) trait (p.MLM = 4.78E-5, Table 2). This observation corresponds with the field response type values (Field.IT) having the highest correlation with seedling IT values against race mixtures (Table 1), likely reflecting the biology that both field response type (Field.IT) and seedling infection type are partially measured by pustule sizes of leaf rust fungus uredinia spores. These results suggest that although field response type (Field.IT) and field severity (Field.SEV) or COI (Field.COI) have high correlations between each other (r > = 0.88), the exact ranks and significance levels of effective loci may differ. Overall, the high number of common loci shared between seedling and field traits suggest that race mixtures can be used to screen for field resistance against the same race mixtures at the seedling stage.

**Fig 7:**
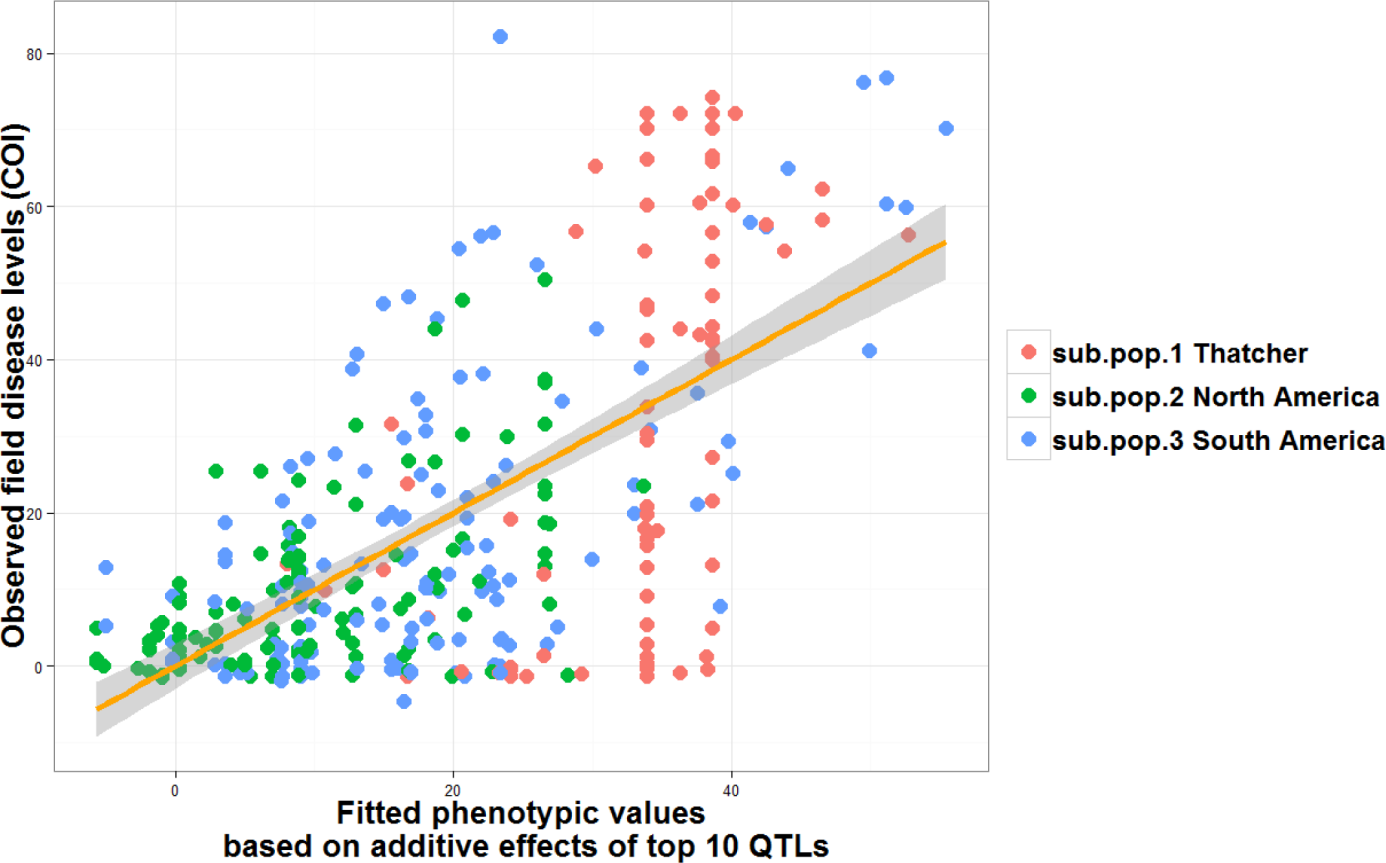
Scatterplot of phenotypic values versus genotypic fitted values using markers representing top ten QTLs for field resistance.

The main contributor genes or loci for seedling resistance included QTLs 6B_3 (likely *Lr9*), 6B_4 (*Lr3*), 5D_1 (*Lr1*), and 2B_2 (likely one of *Lr13*, *Lr16 or Lr23*) (Table 3). The 10 most significant seedling QTLs (Table 3) explained a total of 29.4%, 24.3% and 34.3% phenotypic variations for seedling race 1, seedling race CA1.2, and seedling race mixtures, respectively, after subtracting variance due to population structure. The main contributor loci or genes for field resistance include 4A_1 (QLr.umn-4AS), 1A_3 (QLr.umn-1AL), 2B_2 (likely one of *Lr13*, *Lr16* or *Lr23*) and 5B_2 (likely *QLr*.*cdl-5BL*) [49] and 7B_4 (likely *Lr68*). We plotted the cumulative additive effects of the 10 most significant QTLs (Table 3) in each line of the TCAP leaf rust AM population with their observed phenotype in the field (Fig 7). The selected 10 QTLs for field resistance (measured as COI) (Table 3) explained over 26% of the total phenotypic variation (excluding the amount of contribution derived from population structure). The 10 most significant for field traits (severity and response type) explained 26% and 30% of the phenotypic variations (S4 Fig). Three of the 10 field QTLs (QLr.umn-1AL, QLr.umn-4AS and Q.Lr.cimmyt-4BL) together explained 12% of phenotypic variation (after removing population structure effects). *QLr*.*umn-4AS* is the only QTL whose p.MLM and p.QGLM values both pass the *simpleM* threshold for field resistance. A Haploview [29] of this region is provided (S5 Fig). BLAST search using flanking loci sequence information revealed a possible candidate gene, belonging to the family of ras proteins. One family member of this family was previously identified to be involved in resistance against wheat stripe rust pathogen [53].

We utilized stepwise regression [13, 54] to identify the minimal number of markers that are independently associated with leaf rust resistance for each trait. Our results indicate that, overall, the most significant markers are often included by stepwise regression to account for phenotypic variation (Table 3). However, there are cases where markers with less significance levels were included. For example, the *Lr34* marker (csLv34, Table 2) was associated with the leaf rust severity trait (p = 2.03E-3), but its rank of significance is only at 15^th^ (among all 46 loci). However, it was included as one of the seven markers (IWB19584.1B_1, IWA3151.2A_3, IWA6877.3A_1, IWB59410.4A_1, IWB39735.5B_2, IWB40242.6A_t1, cslv34.7D_1) to account for a significant portion of variation (1.5% out of a total of 25%) in the multiple regression model for leaf severity (Field.SEV), but not for leaf response type (Field.IT). These results suggest that leaf rust severity and response types are probably controlled by different genetic loci, and is consistent with the role of *Lr34* as a gene mostly effective for APR (as response type likely share more common loci with seedling resistance).

Overall, the leaf rust phenotype for field and seedling plants both suggest the presence of resistance alleles in a large percentage of lines within this AM population (Fig 1). The number of favorable alleles and disease resistance are significantly correlated (p<0.05). Our GWAS results confirmed the presence of multiple favorable alleles in a high percentage of individuals within the AM population. We identified a subset of cultivars or lines that possess favorable alleles for 6 of the 7 QTLs obtained through stepwise regression (see above paragraph for regression model components) for field resistance measured by coefficient of infection (COI). As expected, their field evaluation often showed “trace” resistance (Field.Sev < 5, S4 Table). Adult plant leaf rust resistance breeding could potentially be rapidly improved by selecting lines with complimentary resistance genes or QTLs as parents.

## Discussion

### Population Structure and Its Relationship with Leaf Rust Disease Levels and GWAS Results

For field data, the observation that sub-population 1 (Thatcher NILs) was associated with higher disease (Fig 4) was not unexpected. Thatcher is susceptible to leaf rust and the Thatcher near isogenic lines carry single resistance genes that are mostly ineffective against multiple race mixtures as tested in the field. Sub-pop 2 (mostly North American lines) were more resistant than the other two sub-populations. It remains to be explored whether this is partially due to the better adaptation of sub-pop 2 in Northern environments (such as day length), or more likely, because the lines have been selected for leaf rust resistance with the races that are common in North America [55]. The South American lines were selected for inclusion in the panel based on their resistance to predominant races in South America. Sub-population 3 (mostly CIMMYT and South American lines, but some North American lines as well, including lines from MN, North Star Genetics, and Limagrain) has the largest range of phenotypic variation, reflecting its more diverse geographical origins and genetic compositions (lower *Fst* values compared to the other two sub-populations).

Collectively, both origin of the lines and structure analysis based on molecular markers suggest that the true population structures were well captured in our GWAS analysis. It is worth noting that some of the alleles were (nearly) fixed in sub-population 1, as this population consists of almost entirely Thatcher near isogenic lines, which means that they share a significant portion of exactly the same genome. Thus, the presence of favorable marker alleles (particularly in this sub-population) might not indicate the presence of resistant genes. Nonetheless, QTL 5D_1 (*Lr1*) and QTL 6B_4 (*Lr3*) alleles were detected only in a few Thatcher NILs, and our evidence suggests that these are SNPs that are in high LD (or may even be diagnostic) with the gene. We also explored the effects of removing sub-population 1 on GWAS analysis results, and found that the most significant loci were mostly not affected. For example, QTL 5D_1 (Lr1) and 6B_4 (Lr3), 4A_1 (QLr.umn-4AS), 1A_3 (QLr.umn-1AL) were again detected (results not shown), adding further support that these are true loci conferring resistance to the leaf rust disease.

### Comparisons between Models

The difference between the two models (MLM QK and QGLM) used in this study lies in whether Kinship was included into the model analysis. As expected, the p-values derived from QGLM model are generally more significant compared to MLM (QK) model (Table 3), as no kinship relatedness was factored into the model analysis. We further noticed that the p-value differences between MLM (QK) and QGLM models are more pronounced for seedling traits than for field traits (Table 3). It was known that for this panel, many related cultivars or lines possess certain seedling leaf rust resistance genes. For example, the NDSU cultivars Faller and Glenn and the MN cultivar RB07 all possess *Lr21* [56]. Thus, kinship itself could be correlated with the presence or absence of certain seedling leaf rust genes. Correcting for kinship within each sub-population could result in lower statistical power to detect those true QTLs or genes under these circumstances. The QQ plot (S3 Fig) shows that for some traits such as the seedling disease levels against race mixtures, the upper corner dots (higher significance level p-values) curved downwards instead of upwards, possibly indicating that there might be over-correction in the MLM (QK) model analysis. It might make more sense to use p-values derived from QGLM analysis to assist QTL or gene detection under these circumstances. Overall, the QGLM models and MLM (QK) models reveal a similar set of markers. As an exploratory analysis, the G-model revealed QTLs that are often at the same positions of the MLM or QGLM models. The two putatively novel loci (QLr.umn-1AL, QLr.umn-4AS) were repeatedly detected using either one of the models or the G-model, suggesting the robustness of these associations and likelihood that these associations represent relevant *Lr* resistance loci.

### Comparison of Seedling and Field Resistance Genes or QTLs

Although seedling resistance QTLs or genes against race 1 are largely non-overlapping with field resistance loci (Fig 6), we found a high percentage of common loci for field and seedling resistant against race CA1.2 and race mixtures (68 and 80 percent for race CA1.2 and race mixtures respectively) (Fig 6) with consistent marker effects. These results suggest that the seedling resistance genes we detected also contribute to field resistance.

This study also detected some resistance effects in the same regions as the known race non-specific resistance genes (*Lr34*, and possibly *Lr68*) that are effective in the adult plant stage. Gene *Lr34* encodes an ABC transporter and is effective against multiple pathogen species including leaf rust (*Puccinia triticina*), stripe rust (*P*. *striiformis*) and powdery mildew (*Blumeria graminis*) [57]. Our results are consistent with the previous discovery that *Lr34* is more effective in the adult plant stage rather than the seedling stage.

One of the major QTLs for field resistance (QLr.umn-4AS) was detected in every field environment but not at the seedling stage. Similarly, a few other loci (such as 7B_4, 4B_3 and 5B_2, possibly representing *Lr68*, *QLr*.*cimmyt-4BL*, *QLr*.*cdl-5BL*) were also detected for APR but not for seedling resistance. The presence of multiple adult plant APR only loci suggest that field and seedling resistance differ considerably, despite a moderate correlation and the presence of multiple common loci (Table 1 and Fig 5B) between the field and seedling resistance.

### Potential Applications in Future Research

The wheat 90K SNP array is among the highest density genotyping platforms available for wheat researchers [5] and has a much improved coverage of the genome than the 9K SNP array [4]. Various research projects have been published using this genotyping platform [58–61]. The availability of high density consensus maps [5] coupled with the rapid progress in genome-wide sequencing efforts [38, 62] will greatly enhance our ability to dissect important agronomic traits such as leaf rust resistance.

The QTLs or genes identified or validated in this study were associated with sequence based markers which could be more efficiently anchored to reference genomes [62] than traditional markers such as simple sequence repeats (SSRs). Assays such as KASP [63, 64] can be developed based on closely linked SNP markers (such as *QLr*.*umn-4AS*, *5D_1*.*Lr1*, *7B_4*.*Lr3* loci) to provide more high-throughput genotyping and marker assisted breeding. Contextual genome sequences around target SNPs might provide direct insights into the genetic composition of trait of interest. Accessions with a high percentage of leaf rust resistance alleles could serve as parental breeding lines to enable more efficient breeding, especially for adult plant resistance.

## Conclusions

We conducted a genome-wide association study on a population consisting of mostly breeding lines with known seedling or adult plant resistance. This study is among the first GWAS studies that utilizes the wheat iSelect 90K SNP array to explore leaf rust resistance QTLs. A large percentage of lines were associated with multiple resistance alleles or QTLs for leaf rust resistance. The 10 most significant QTLs accounted for 24–34% of phenotypic variation for each trait analyzed. Compared to single races, leaf rust reaction to race mixtures in the seedling test best resembled field resistance, suggesting that field resistance can be partially screened in the seedling stage using race mixtures. Identification of novel QTLs (such as *QLr*.*umn-1AL*, *QLr*.*umn-4AS*) with field resistance against leaf rust could enhance our understanding of leaf rust resistance and provide new resources of leaf rust resistance. Identification of a subset of lines with a high percentage of favorable alleles (based on SNP marker information) may serve as valuable parental materials for further resistance breeding.

## Supporting Information

S1 Fig Marker density among wheat chromosomes. Number of unique markers is defined as the number of markers that are at different positions of the consensus map (Wang et al 2014).
(PPTX)

S2 Fig Flowering locus detected within this leaf rust AM panel. (PPTX)

S3 Fig QQ plots for seedling and field traits. (PPTX)

S4 Fig Scatterplots of fitted versus observed values based on the genotype of most significant 10 loci for Field.SEV and Field.IT traits. (PPTX)

S5 Fig QLr.umn.4AS LD region visualized in Haploview. Picture showing relative position and degree of LD in the region.
(PPTX)

S1 Table A list of 333 marker p-values, R^2^ and effects based on MLM analysis using TASSEL. (CSV)

S2 Table A list of 333 marker p-values, R^2^ and effects based on MLM analysis using GAPIT. (CSV)

S3 Table A list of 107 marker p-values, effects based on G-model analysis. (XLSX)

S4 Table A subset of cultivars or lines that are superior based on stepwise regression models. (XLSX)
