# Supplementary material for: Genome Wide Association Study of Seedling and Adult Plant Leaf Rust Resistance in Elite Spring Wheat Breeding Lines
Source: PLoS One. 2016 Feb 5;11(2):e0148671. doi: 10.1371/journal.pone.0148671 (PMC4744023; doi:10.1371/journal.pone.0148671)
Supplement: S1 Fig — Number of unique markers is defined as the number of markers that are at different positions of the consensus map (Wang et al 2014). (PPTX) [file pone.0148671.s001.pptx]

## Slide 1
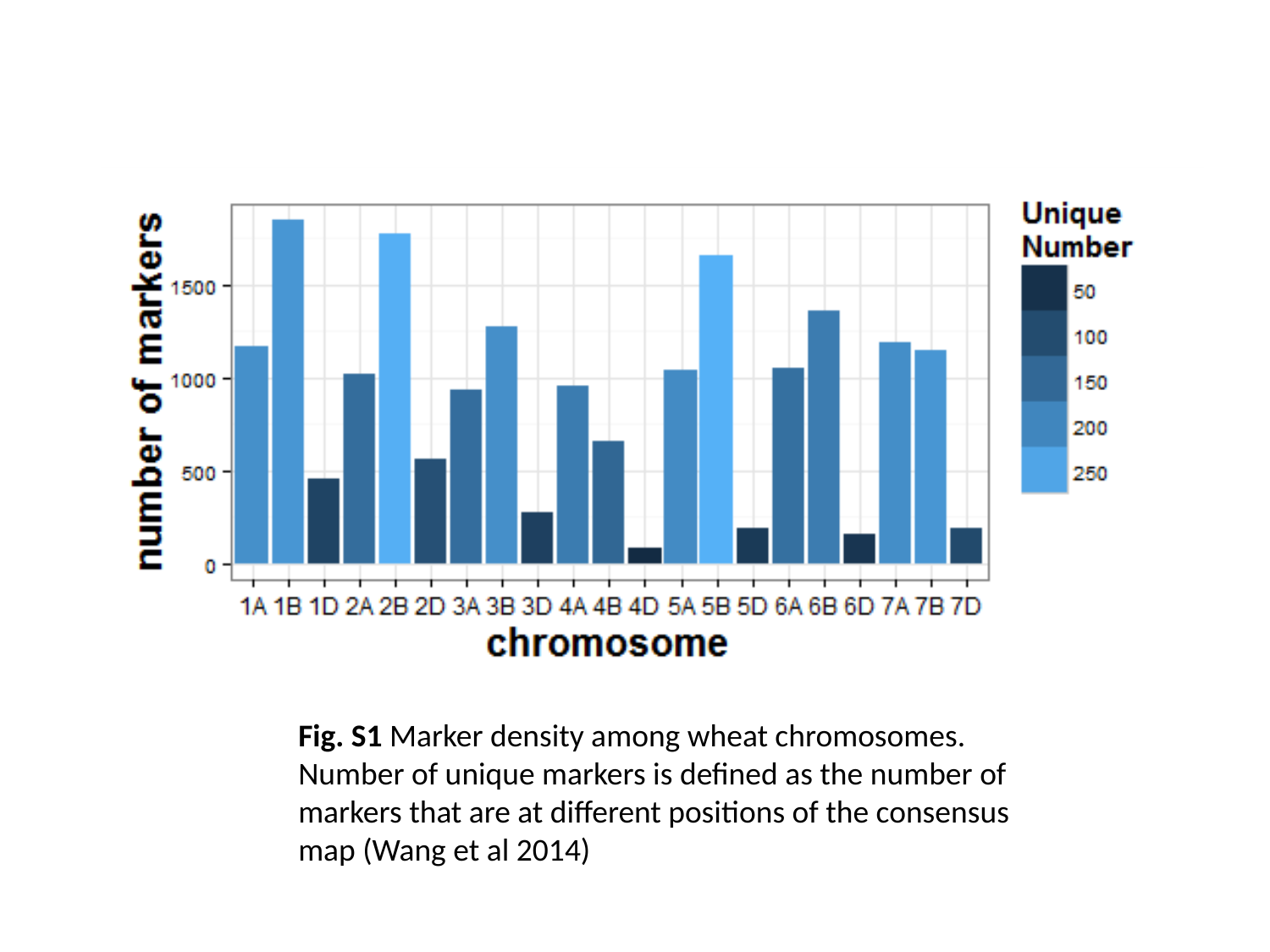

Fig. S1 Marker density among wheat chromosomes. Number of unique markers is defined as the number of markers that are at different positions of the consensus map (Wang et al 2014)
