# Supplementary figures and images for: Genome Wide Association Study of Seedling and Adult Plant Leaf Rust Resistance in Elite Spring Wheat Breeding Lines
Source: PLoS One. 2016 Feb 5;11(2):e0148671. doi: 10.1371/journal.pone.0148671 (PMC4744023; doi:10.1371/journal.pone.0148671)

## Slide 1
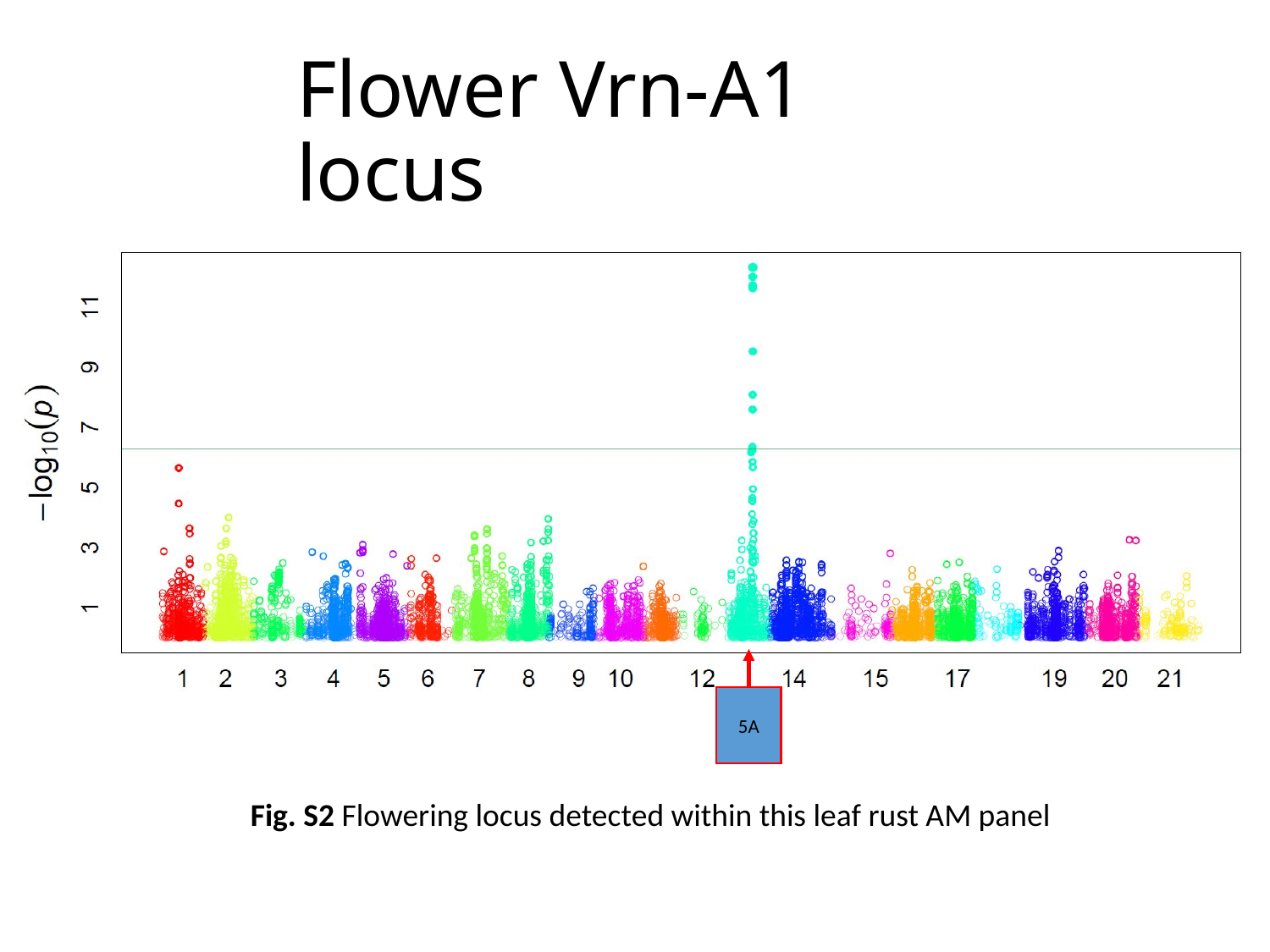

# Flower Vrn-A1 locus
5A
Fig. S2 Flowering locus detected within this leaf rust AM panel

Supplement: S2 Fig — (PPTX) [file pone.0148671.s002.pptx]

## Slide 1
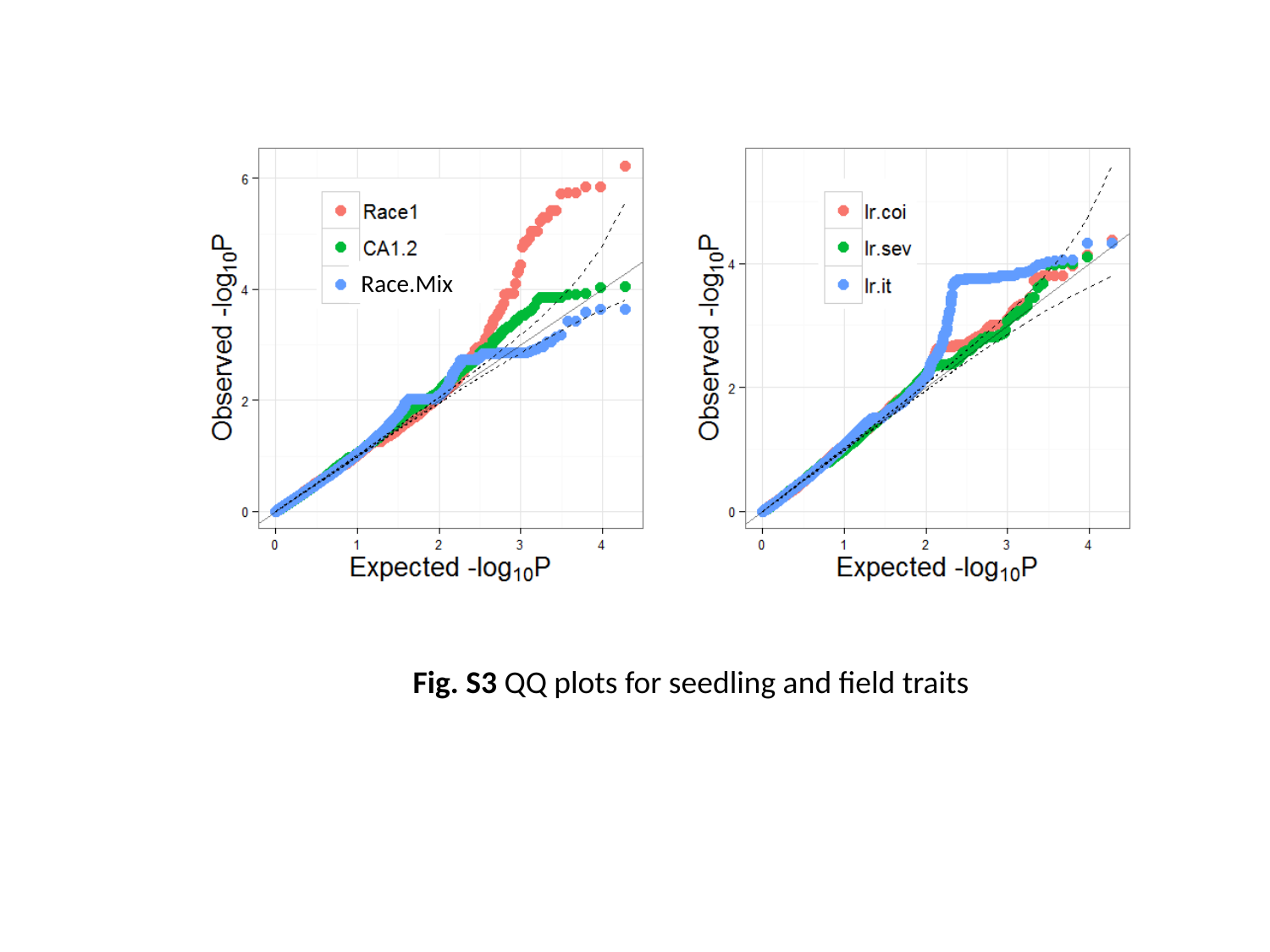

Race.Mix
Fig. S3 QQ plots for seedling and field traits

Supplement: S3 Fig — (PPTX) [file pone.0148671.s003.pptx]
