# Supplementary material for: Genome Wide Association Study of Seedling and Adult Plant Leaf Rust Resistance in Elite Spring Wheat Breeding Lines
Source: PLoS One. 2016 Feb 5;11(2):e0148671. doi: 10.1371/journal.pone.0148671 (PMC4744023; doi:10.1371/journal.pone.0148671)
Supplement: S4 Fig — (PPTX) [file pone.0148671.s004.pptx]

## Slide 1
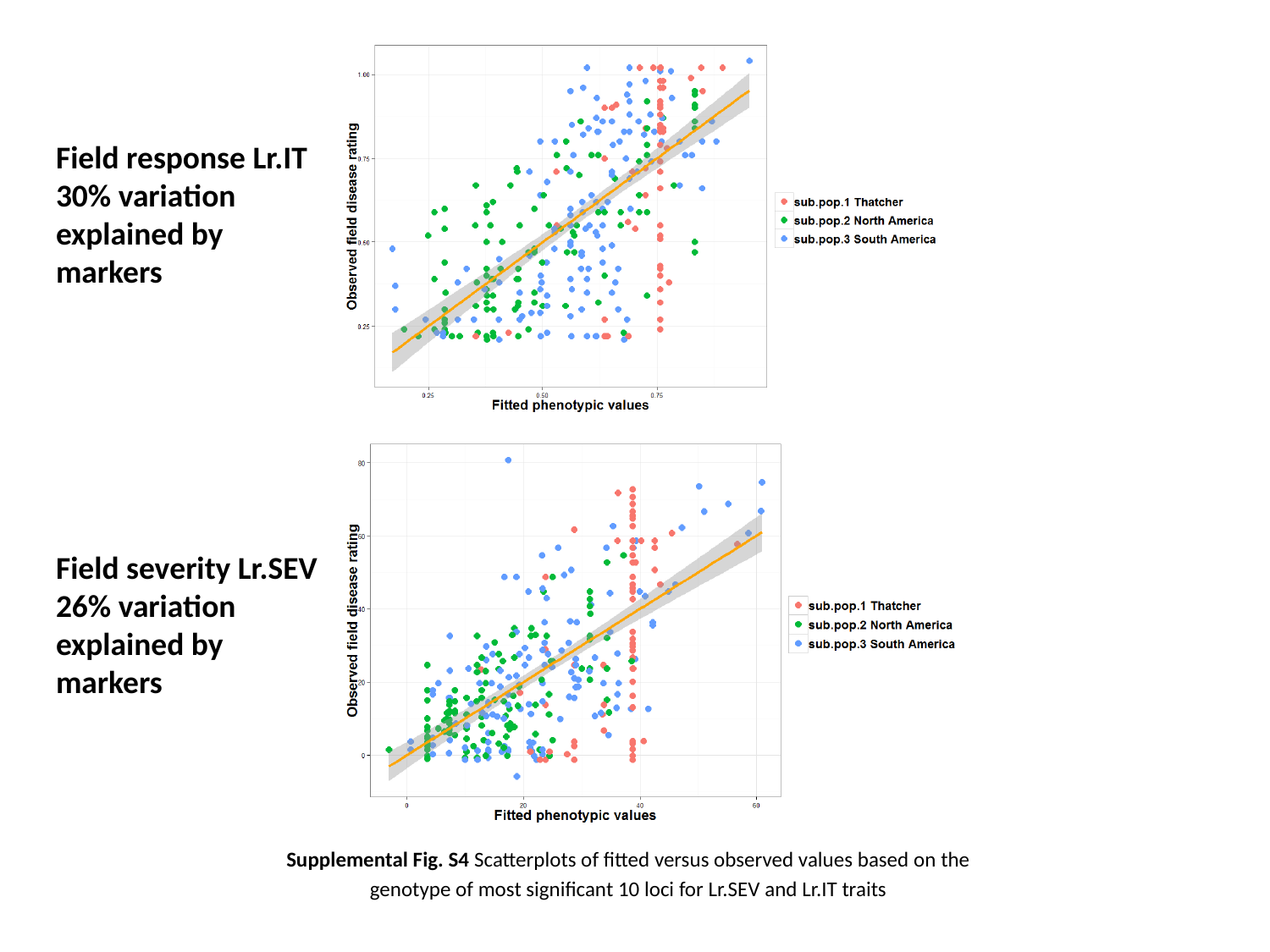

Field response Lr.IT
30% variation explained by markers
Field severity Lr.SEV
26% variation explained by markers
Supplemental Fig. S4 Scatterplots of fitted versus observed values based on the genotype of most significant 10 loci for Lr.SEV and Lr.IT traits
