# Supplementary material for: Genome Wide Association Study of Seedling and Adult Plant Leaf Rust Resistance in Elite Spring Wheat Breeding Lines
Source: PLoS One. 2016 Feb 5;11(2):e0148671. doi: 10.1371/journal.pone.0148671 (PMC4744023; doi:10.1371/journal.pone.0148671)
Supplement: S5 Fig — Picture showing relative position and degree of LD in the region. (PPTX) [file pone.0148671.s005.pptx]

## Slide 1
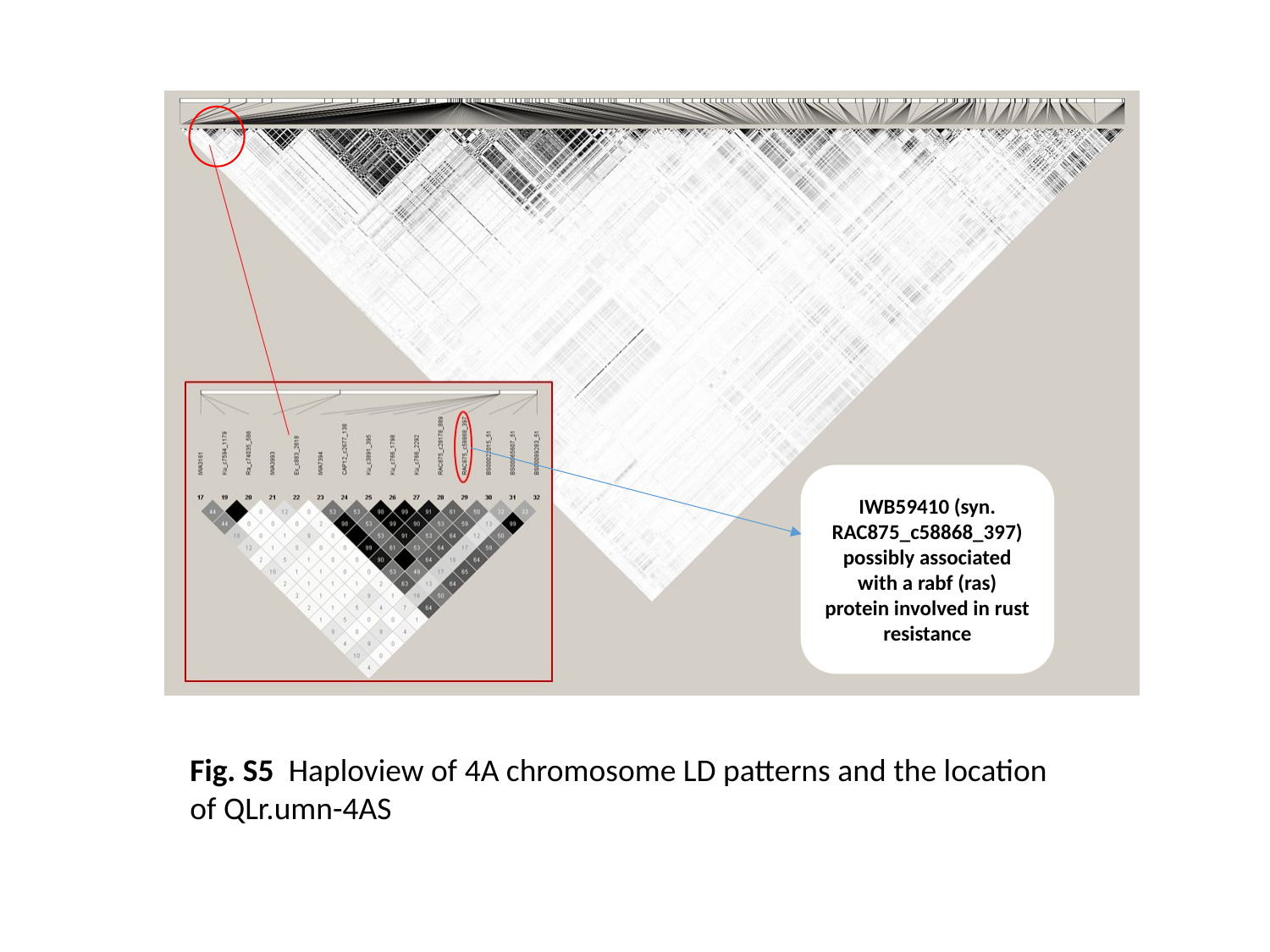

IWB59410 (syn. RAC875_c58868_397) possibly associated with a rabf (ras) protein involved in rust resistance
Fig. S5 Haploview of 4A chromosome LD patterns and the location of QLr.umn-4AS
